# Neoaortic Regurgitation Detected by Echocardiography After Arterial Switch Operation: A Systematic Review and Meta-Analysis

**DOI:** 10.1016/j.jacadv.2024.100878

**Published:** 2024-03-07

**Authors:** Xander Jacquemyn, Jef Van den Eynde, Art Schuermans, Roel L.F. van der Palen, Werner Budts, David A. Danford, William J. Ravekes, Shelby Kutty

**Affiliations:** a Department of Pediatrics, Helen B. Taussig Heart Center, Johns Hopkins Hospital, Baltimore, Maryland, USA; b Department of Cardiovascular Sciences, KU Leuven, Leuven, Belgium; c Program in Medical and Population Genetics and Cardiovascular Disease Initiative, Broad Institute of Harvard and MIT, Cambridge, Massachusetts, USA; d Cardiovascular Research Center and Center for Genomic Medicine, Massachusetts General Hospital, Boston, Massachusetts, USA; e Division of Pediatric Cardiology, Department of Pediatrics, Leiden University Medical Center, Leiden, the Netherlands; f Congenital and Structural Cardiology, Department of Cardiovascular Sciences, UZ Leuven, KU Leuven, Leuven, Belgium

**Keywords:** arterial switch operation, neoaortic regurgitation, neoaortic root dilatation, transposition of the great arteries

## Abstract

**Background:**

Neoaortic root dilatation (NeoARD) and neoaortic regurgitation (NeoAR) are common sequelae following the arterial switch operation (ASO) for transposition of the great arteries.

**Objectives:**

The authors aimed to estimate the cumulative incidence of NeoAR, assess whether larger neoaortic root dimensions were associated with NeoAR, and evaluate factors associated with the development of NeoAR during long-term follow-up.

**Methods:**

Electronic databases were systematically searched for articles that assessed NeoAR and NeoARD after ASO, published before November 2022. The primary outcome was NeoAR, classified based on severity categories (trace, mild, moderate, and severe). Cumulative incidence was estimated from Kaplan-Meier curves, neoaortic root dimensions using Z-scores, and risk factors were evaluated using random-effects meta-analysis.

**Results:**

Thirty publications, comprising a total of 6,169 patients, were included in this review. Pooled estimated cumulative incidence of ≥mild NeoAR and ≥moderate NeoAR at 30-year follow-up were 67.5% and 21.4%, respectively. At last follow-up, neoaortic Z-scores were larger at the annulus (mean difference [MD]: 1.17, 95% CI: 0.52-1.82, *P* < 0.001; MD: 1.38, 95% CI: 0.46-2.30, *P* = 0.003) and root (MD: 1.83, 95% CI: 1.16-2.49, *P* < 0.001; MD: 1.84, 95% CI: 1.07-2.60, *P* < 0.001) in patients with ≥mild and ≥moderate NeoAR, respectively, compared to those without NeoAR. Risk factors for the development of any NeoAR included prior pulmonary artery banding, presence of a ventricular septal defect, aorto-pulmonary mismatch, a bicuspid pulmonary valve, and NeoAR at discharge.

**Conclusions:**

The risks of NeoARD and NeoAR increase over time following ASO surgery. Identified risk factors for NeoAR may alert the clinician that closer follow-up is needed. (Risk factors for neoaortic valve regurgitation after arterial switch operation: a meta-analysis; CRD42022373214).

The arterial switch operation (ASO) for transposition of the great arteries (TGA) was first successfully performed in 1976 by Jatene et al.^1^ Following modifications made by Lecompte et al,^2^ the ASO has become the standard of care for restoring appropriate physiology and anatomy in TGA. As early mortality and morbidity following the ASO have diminished, the TGA population has become substantially older, and complications, including right ventricular outflow tract obstruction, pulmonary artery stenosis, neoaortic root dilatation (NeoARD), and neoaortic regurgitation (NeoAR), have been observed later in the post-ASO course.^3^ To prevent the threats to health associated with these complications, it is expected that there will be an increasing need for neoaortic valve surgery (NeoAVS) and neoaortic root reoperation among older TGA patients.^3^ Yet, data on the long-term effects of ASO on neoaortic growth and function are inconsistent. Some studies have shown stabilization of NeoARD and NeoAR over time, while others have described significant ongoing late progression. Moreover, risk factors associated with late NeoAR and NeoARD vary considerably across reports.^4^^,^^5^

## Methods

The protocol for this meta-analysis and systematic review was finalized a priori and registered with PROSPERO (CRD42022373214). We followed the Preferred Reporting Items for Systematic Reviews and Meta-Analyses statement. Ethical approval/institutional review board approval was not required. Additional Supplemental Methods are available in the Supplemental Appendix.

### Data sources, searches, and eligibility criteria

Systematic literature searches were conducted in PubMed, EMBASE, Scopus, and the Cochrane Library electronic databases up to November 1, 2022. Studies were included if the following criteria were fulfilled:

1. The population comprised patients with TGA who underwent ASO;
2. Primary outcomes studied included NeoAR, NeoARD, and/or NeoAVS;
3. Longitudinal follow-up data were available, and estimates for the outcomes of interest were reported up to at least 10 years post-ASO.

### Study selection and data extraction

Studies were selected by 2 independent reviewers (X.J. and A.S.). When there was disagreement, the final decision to include or exclude the study was made in consensus. The Risk of Bias in Non-Randomized Studies of Interventions tool was systematically applied to assess all included studies for risk of bias.^6^ The studies and their characteristics were classified by 2 independent reviewers (X.J. and A.S.).

### Outcomes

The primary outcome of interest in this study was NeoAR, while secondary outcomes include NeoARD and NeoAVS. NeoAR was collected as a semiquantitative/qualitative grade (including none, trace, mild, moderate, and severe) from original studies. NeoARD was defined as a Z-score ≥2.5 (or extracted as per original institutional definitions). NeoAVS was defined as the need for reintervention for neoaortic root dilation or neoaortic valve regurgitation, including valve repair or replacement.

### Statistical analysis

To estimate the cumulative incidence of NeoAR and other secondary adverse outcomes, we employed the “curve approach” reconstructing individual patient data based on published Kaplan-Meier graphs from included studies using a 2-stage approach.^7^ Kaplan-Meier plots were digitized to raw data coordinates using an online web-based plot digitizer software (Web Plot Digitizer, Version 4.6), and individual patient data was reconstructed from the raw data coordinates using the R package “IPDfromKM” (version 0.1.10).^8^ Risk factors for NeoAR during follow-up from individual studies were pooled using random-effects models. Time-to-event data were analyzed using a Cox frailty model with a robust variance estimator. Risk factors were incorporated as fixed-effects, and the study factor was included as a γ frailty term (random-effects). In addition, the random-effect results were reanalyzed using fixed-effects models to explore whether this yielded potential variations in the summary inferences. All analyses were completed with R Statistical Software (version 4.2.1, Foundation for Statistical Computing).

## Results

### Study selection and characteristics

**Figure 1:**
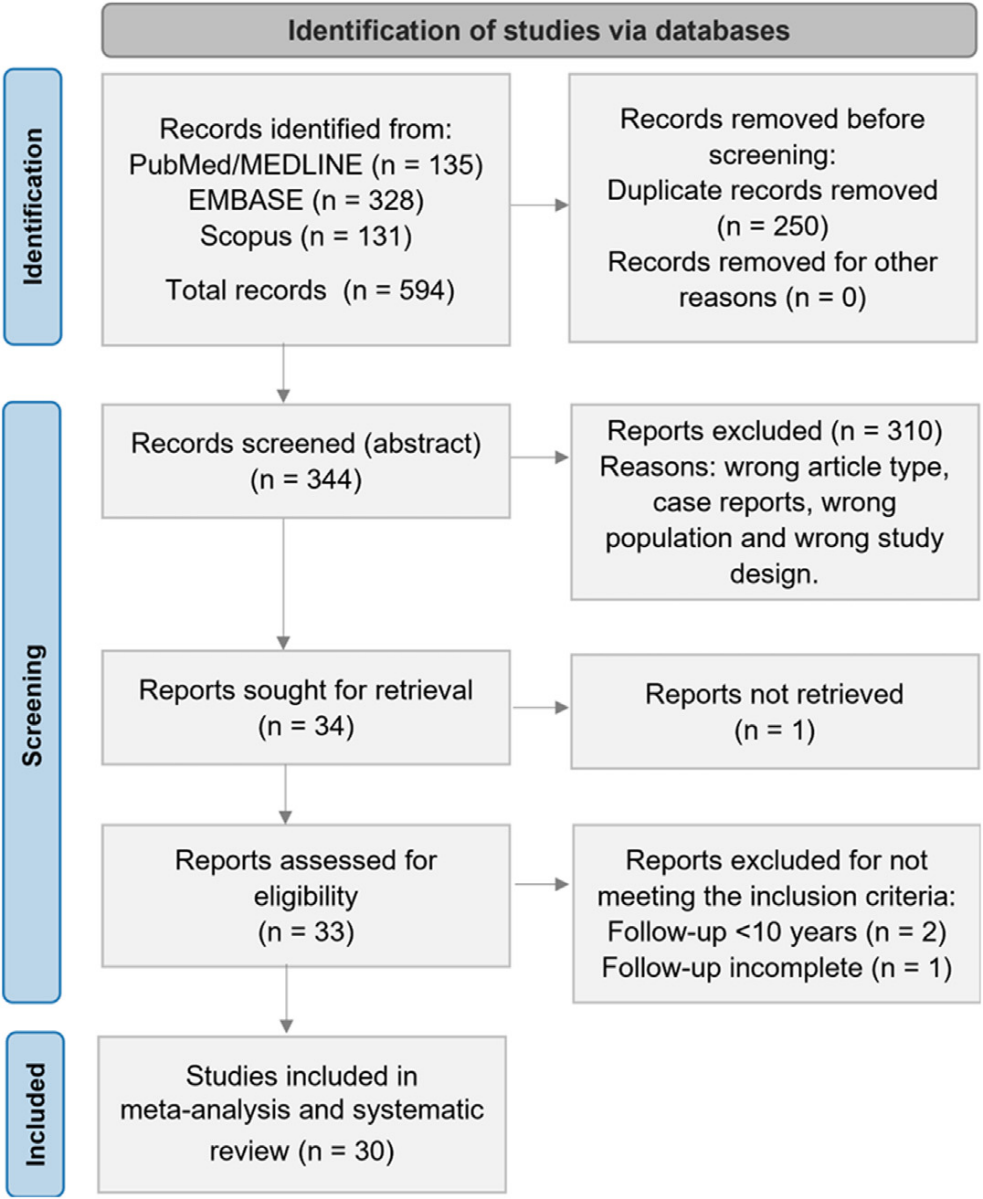
**PRISMA Flow Diagram of Studies Included in Data Search** PRISMA = Preferred Reporting Items for Systematic Reviews and Meta-Analyses.

Our initial search yielded 344 unique citations (Supplemental Table 1); among these, 30 fulfilled our eligibility criteria (Figure 1).^4^^,^^5^^,^^9^, ^10^, ^11^, ^12^, ^13^, ^14^, ^15^, ^16^, ^17^, ^18^, ^19^, ^20^, ^21^, ^22^, ^23^, ^24^, ^25^, ^26^, ^27^, ^28^, ^29^, ^30^, ^31^, ^32^, ^33^, ^34^, ^35^, ^36^ Characteristics of each study and their participants are shown in Table 1. The 30 included studies comprised a total of 6,169 patients and were conducted in 13 different countries across 3 continents. All studies had a retrospective observational design. One included data from multiple centers.^31^ The proportion of cases in which the Lecompte procedure was performed was reported in 10 studies (range 91%-100%). Coronary artery reimplantation techniques varied greatly between centers, and only 12 studies disclosed frequencies of surgical methods used, most commonly (modified) trap door techniques, button techniques, or a combination of both. Less frequently, some studies favored direct coronary anastomosis. In rare cases, other techniques such as the Imai, Yacoub, or aortic sinus pouch techniques were used. Qualitative assessment of the studies with the Risk of Bias in Non-Randomized Studies of Interventions tool demonstrated several concerns regarding confounding factors, missing data, and bias in measurement of outcomes. Thus, the overall internal validity of the analysis was considered moderate risk of bias (Supplemental Figure 1).

**Table 1:** Baseline Characteristics of Included Studies Reporting on Occurrence of Neoaortic Regurgitation

First Author	Year	Location	Centers	Sample Size (n)	Time Period	Male, n (%)	Age at ASO, d	Weight at ASO, kg	VSD, n (%)	TBA, n (%)	AAA, n (%)	BPV, n (%)	NeoAR, n (%)	Follow-up (y)
Bové et al^19^	2008	Belgium	S	93	1993-2006	66 (71)	8	3.47	31 (33.3)	8 (8.6)	12 (12.9)	NA	13 (14)	4.8 ± 3.9
Co-Vu et al^30^	2013	USA	S	124	1984-2007	85 (29)	6 (3-1,800)	NA	36 (49)	NA	14 (19)	7 (10)	17 (14)	7.2 (1-23)
Formigari et al^31^	2003	Italy	M	173	1987-2001	86 (50)	8 (2-344)	3.4 ± 9.6	33 (19)	4 (2.3)	8 (4.6)	6 (3.5)	61 (35)	8.2 (0.6-11.2)
Hutter et al^32^	2001	the Netherlands	S	144	1977-2000	NA	8 (1-1,878)	NA	47 (33)	16 (11.1)	9 (6.3)	6 (4.2)	5 (3.5)	8.7 (0.1-22.5)
Irwin et al^33^	2021	USA	S	278	1989-2018	174 (63)	8.2 ± 2.8	NA	139 (50)	NA	NA	67 (24)^a^	4 (4.2)	11.3 (0.02-30.3)
Jeon et al^34^	2022	Korea	S	75	1997-2018	39 (52)	12 [7-20]	3.2 [2.8-3.6]	45 (60)	13 (17.3)	0 (0)	15 (25)^a^	NA	9.9 (0.37-22.3)
Lange et al^35^	2008	Germany	S	479	1983-2006	NA	11 (2-4,928)	3.5 (2.1-57)	141 (29.4)	NA	43 (9)	21 (4)	41 (8.7)	9.3 (0-22.6)
Lim et al^26^	2013	Korea	S	220	1987-2011	NA	13 (0-1,768)	3.52 (1.7-19)	90 (40.9)	17 (7.7)	NA	NA	78 (38.0)	8.6 (0-23.1)
Lo Rito et al^4^	2015	United Kingdom	S	362	1988-1998	264 (73)	8 (1-3,905)	3.45 (1.8-22.4)	151 (41.7)	30 (8.3)	39 (10.8)	NA	97 (45.8)	16 [12-18.2]
Losay et al^27^	2001	France	S	1,095	1982-1999	NA	29 ± 93	3.5 ± 1.3	258 (23.6)	67 (6.1)	111 (10.1)	NA	165 (15.5)	4.9 ± 3.4
Losay et al^36^	2006	France	S	1,156	1982-2000	NA	32.6 ± 142	3.5 ± 1.9	269 (23.3)	72 (6.2)	113 (9.8)	NA	172 (14.9)	6.25 (0-20)
Ma et al^9^	2016	China	S	583	2003-2013	NA	233 ± 654	3.1 ± 5.3	313 (53.7)	0 (0)	13 (2.2)	14 (2.4)	56 (10.4)	3.83 (0.67-10)
Marino et al^10^	2006	USA	S	82	1984-1997	54 (66)	5 (1-1,825)	NA	30 (37)	NA	8 (9.7)	NA	69 (84.1)	8.8 (4.1-16.4)
Martins et al^11^	2018	Brazil	S	127	1997-2015	90 (70.8)	NA	NA	43 (33.9)	NA	7 (5.5)	NA	37 (29.1)	7.4 ± 4.7
Martins et al^12^	2019	France	S	157	2010-2017	108 (69)	14.9 ± 30.9	NA	41 (26)	NA	20 (13)	8 (5.0)	35 (22)	14.9 ± 4.6
McMahon et al^13^	2004	USA	S	119	1986-2001	75 (63)	2 (2-37)	NA	36 (30.3)	10 (8.4)	9 (7.6)	0 (0.0)	32 (27)	5.42 (1-15)
Michalak et al^14^	2010	Poland	S	161	1991-2008	116 (72)	9.79 ± 10.67	3.5 ± 1.3	52 (32)	NA	0 (0)	12 (7)	75 (47)	12.6 (10-18)
Michalak et al^15^	2013	Poland	S	172	1992-2011	122 (70)	9.68 ± 9.45	3.37 ± 0.56	51 (28)	NA	12 (7)	NA	85 (49)	13.5 ± 2.4
Michalak et al^16^	2020	Poland	S	56	1991-2018	NA	NA	NA	NA	NA	NA	NA	30 (53.6)	19.8 (17.9-23)
Muneuchi et al^17^	2022	Japan	S	45	1986-2019	34 (75.5)	44.2 ± 39.8	3.51 ± 0.86	17 (37.7)	NA	9 (20)	NA	8 (17.8)	21.7 ± 2.0
Nakayama et al^5^	2019	Japan	S	469	1982-2016	NA	30.5 (3.1-2,486)	3.5 (2.1-20.4)	140 (29.9)	NA	32 (6.8)	9 (1.9)	41 (8.6)	19.0 (0.1-35.2)
Oda et al^29^	2012	Japan	S	387	1984-2010	NA	19	NA	98 (25.3)	NA	52 (13.4)	7 (1.8)	29 (7.5)	10 ± 7.4
Oda et al^18^	2019	Japan	S	145	1984-2015	101 (69.7)	NA	NA	46 (31.7)	NA	19 (13.1)	1 (0.7)	21 (14.5)	13.8 (11.9-17.9)
Prifti et al^20^	2002	Italy	S	134	1990-2001	79 (59)	NA	4.8 ± 3.7	39 (29.1)	8 (6.0)	20 (15)	NA	10 (7.5)	3.4 (0.67-12)
Puras et al^28^	2014	Spain	S	155	1985-2010	NA	13 (4-4,015)	NA	46 (29.7)	5 (3.2)	7 (4.5)	NA	43 (28)	6 (0-25)
Schwartz et al^21^	2004	USA	S	335	1981-2000	192 (66.9)	6 (0-2,847)	3.5 (1.5-31.9)	151 (45.1)	NA	47 (14.0)	10 (3.0)	17 (5.1)	>5 (0-18)
van der Palen et al^37^	2019	the Netherlands	S	345	1977-2015	229 (66.4)	8 (0-219)	NA	89 (25.8)	26 (7.5)	24 (7.0)	21 (6.1)	33 (9.6)	12.2 (1-39)
W.K. Jhang et al^25^	2012	Korea	S	240	1991-2010	166 (69.1)	11 (0-1,213)	3.4 (1.3-18.8)	100 (41.6)	18 (7.5)	16 (6.6)	12 (5.0)	6 (2.5)	6.6 (1-19.5)
Walter et al^23^	2010	Germany	S	324	1987-2008	215 (66.3)	6.3 ± 0.4	3.3 ± 0.5	NA	NA	6 (3)	NA	17 (5.2)	14.4 (1-17.8)
Wang et al^24^	2022	China	S	185	2006-2022	131 (70.8)	24 ± 1,240	3.54 (2.2-7.3)	64 (34.6)	21 (11.4)	11 (5.95)	35 (18.67)	19 (11.5)	7.4 (0-15.6)

Values presented as mean ± SD, median (range), median [IQR], and n/N (%) according to originally published data.

AAA = aortic arch anomalies; ASO = arterial switch operation; BPV = bicuspid pulmonary valve; NeoAR = neoaortic regurgitation; TBA = Taussig-Bing Anomaly; VSD = ventricular septal defect.

a Bicuspid pulmonary valve groups were matched.

### Cumulative incidence of neoaortic regurgitation, root dilatation, and valve surgery

**Figure 2:**
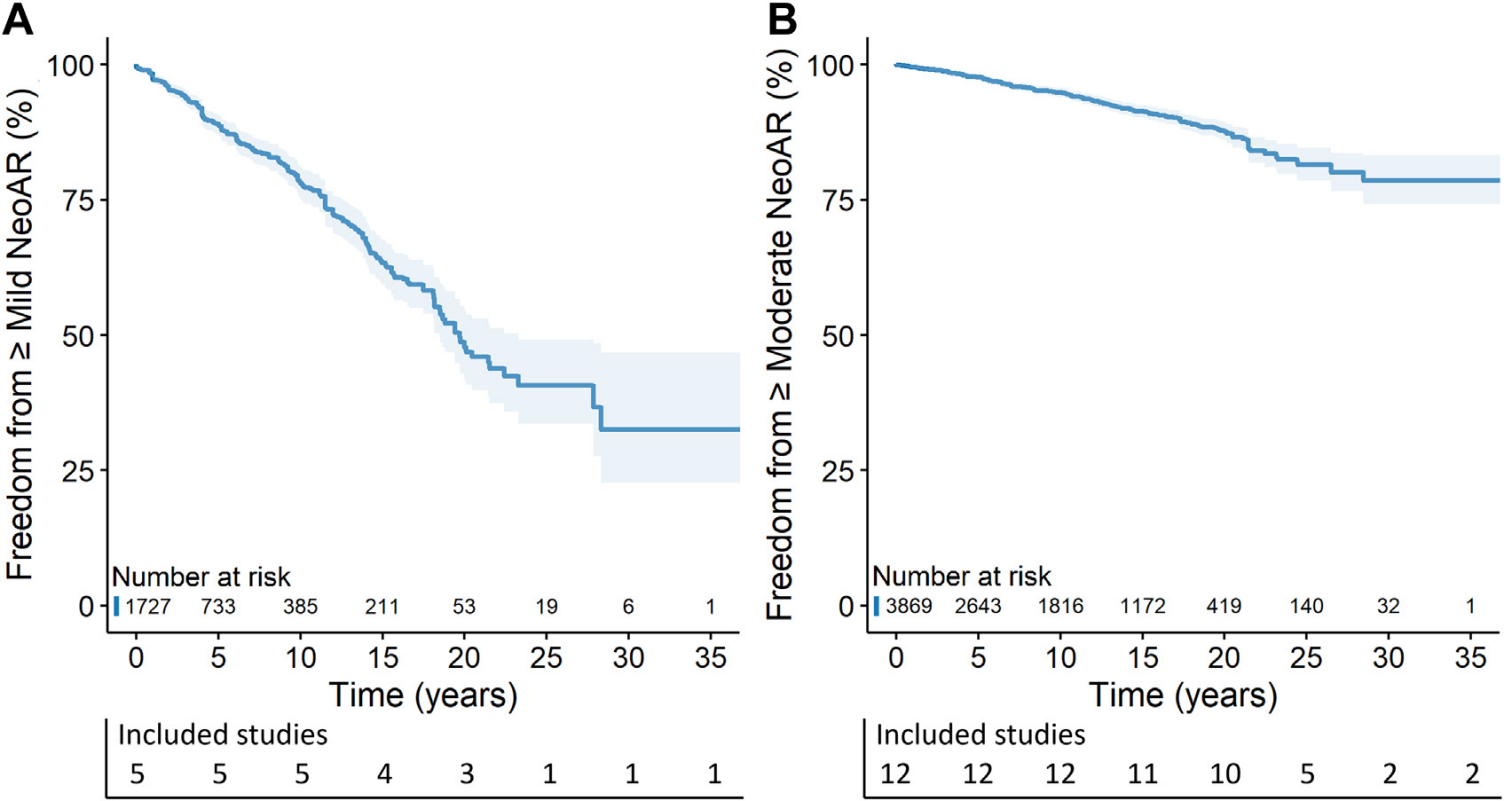
**Cumulative Risk of NeoAR During Follow-Up After ASO for TGA** Freedom from (A) ≥mild NeoAR and (B) ≥moderate NeoAR during long-term follow-up after ASO. ASO = arterial switch operation; NeoAR = neoaortic regurgitation; TGA = transposition of the great arteries.

**Figure 3:**
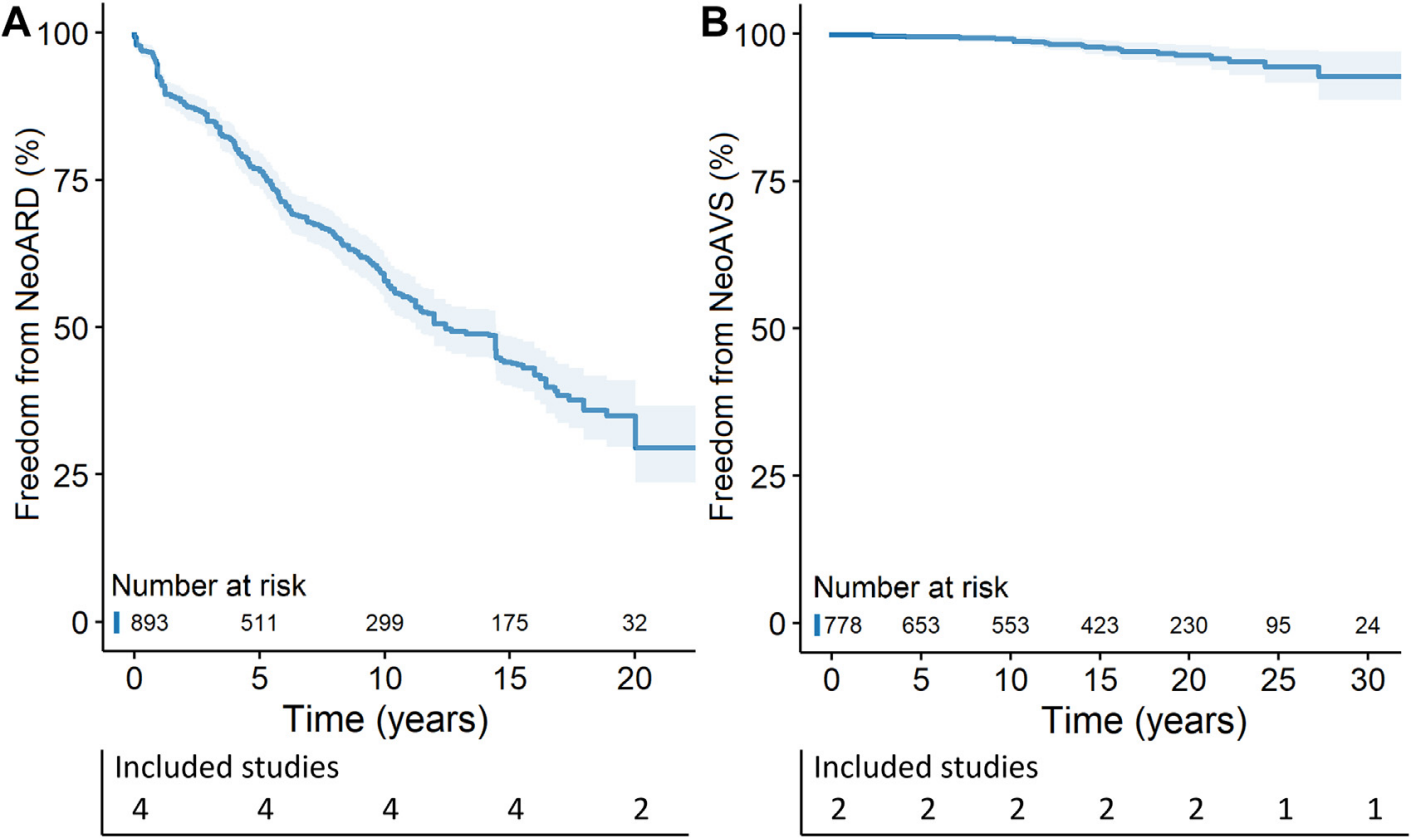
Cumulative Risk of NeoARD and NeoAVS During Follow-Up After ASO for TGA Cumulative risk of (A) NeoARD and (B) NeoAVS during follow-up. NeoARD as defined by the individual studies, ranging between Z-score ≥2.5 and ≥4. ASO = arterial switch operation; NeoARD = neoaortic root dilatation; NeoAVS = neoaortic valve surgery; TGA = transposition of the great arteries.

Prevalence of NeoAR at final echocardiographic follow-up varied greatly among studies, ranging from 2.5% to 84.1%. Most studies used a quantitative or semiquantitative approach to grade NeoAR, which was then converted to a qualitative grade for interpretation and analysis (Supplemental Table 2). Five studies^15^^,^^19^^,^^26^^,^^27^^,^^37^ presented cumulative incidence of ≥mild NeoAR, including 1,727 patients (Figure 2A). Survival free from ≥mild NeoAR was 78.1%, 47.7%, and 32.5% at 10, 20, and 30 years after ASO, respectively. Twelve studies^4^^,^^5^^,^^9^^,^^21^^,^^25^^,^^26^^,^^28^, ^29^, ^30^^,^^33^^,^^35^^,^^37^ presented cumulative incidence of ≥ moderate NeoAR, including 3,869 patients (Figure 2B). Survival free from ≥moderate NeoAR was 94.8%, 87.8%, and 78.6% at 10, 20, and 30 years after ASO, respectively. A total of 4 studies^21^^,^^24^^,^^30^^,^^33^ presented cumulative incidence of NeoARD (definition ranging between Z-score ≥2.5 and ≥4) including 893 patients (Figure 3A). Freedom from NeoARD at 10, 15, and 20 years after ASO was 57.9%, 44.1%, and 34.9%, respectively. A summary of the proportion of NeoAVS during follow-up and the respective performed procedures are presented in Supplemental Table 3. Two studies^4^^,^^5^ reported cumulative incidence of NeoAVS including 778 patients (Figure 3B). NeoAVS-free survival at 10, 20, and 30 years after ASO was 99.4%, 96.5%, and 93.0%, respectively.

### Association of neoaortic root Z-scores with neoaortic regurgitation at follow-up

To determine whether neoaortic root dimensions at echocardiographic follow-up (median 10.5 years, range 0.1-25 years) were associated with NeoAR, we compared these among patients who developed NeoAR and those who did not. We found that increasing neoaortic Z-scores were significantly higher both at the annulus and the root in patients with ≥ mild NeoAR (MD: 1.17 [95% CI: 0.52-1.82], *P* < 0.001; and MD: 1.83 [95% CI: 1.16-2.49], *P* < 0.001, respectively), while no significant difference was observed at the sinotubular junction (MD: 0.47 [95% CI: -0.25-1.19], *P* = 0.202). Additionally, neoaortic Z-scores were significantly higher both at the annulus and the root in patients with ≥moderate NeoAR (MD: 1.38, 95% CI: 0.46-2.30, *P* = 0.003; and MD: 1.84, 95% CI: 1.07-2.60, *P* < 0.001, respectively) (Table 2, Supplemental Figures 2A to 2C and 5).

**Table 2:** Meta-Analysis of Associations With Neoaortic Regurgitation and Risk Factors for Development of Neoaortic Regurgitation: Summary of Results

AR^a^	Association With NeoAR	Effect Size				Heterogeneity		Sensitivity
		Studies (n)	Point Estimate	95% CI	*P* Value	*I*^2^ (%)	*P* Value	Significant on Fixed-Effect
≥mild	Neoaortic Annulus Z-score	4	1.17^b^	0.52-1.82	<0.001	45	0.14	✔
	Neoaortic Root Z-score	4	1.83^b^	1.16-2.49	<0.001	62	0.05	✔
	Neoaortic STJ Z-score	2	0.47^b^	−0.25 to 1.19	0.202	0	0.62	✗
	Baseline risk factors							
≥trace	Prior PAB	9	2.83^c^	1.34-5.96	0.006	49	0.05	✔
	AAA	4	1.46^c^	0.08-26.93	0.811	77	<0.01	✔
	VSD	8	1.82^c^	1.07-3.10	0.027	63	<0.01	✔
	TBA	4	2.66^c^	0.46-15.48	0.279	54	0.09	✔
	Male sex	5	1.00^c^	0.36-2.76	0.982	70	<0.01	✗
	NeoAR at discharge	4	5.64^c^	3.62-8.79	<0.001	0	0.39	✔
	Ao/PA discrepancy	5	2.25^c^	1.44-3.51	<0.001	7	0.37	✔
	BPV	6	2.07^c^	0.71-6.00	0.183	69	<0.01	✔
	Age at ASO (d)	4	8.65^b^	−8.16 to 25.46	0.318	38	0.19	✗
≥moderate	Prior PAB	5	2.56^d^	1.24-5.29	0.011	12	0.34	✔
	VSD	4	1.85^d^	0.68-5.01	0.229	49	0.11	✔
	TBA	3	2.40^d^	0.32-17.83	0.400	50	0.13	✔
	LVOTO	3	2.94^d^	0.09-95.89	0.556	85	<0.01	✗
	Ao/PA discrepancy	4	3.72^d^	0.40-34.70	0.251	85	<0.01	✔
	BPV	5	1.96^d^	1.01-3.81	0.047	18	0.30	✔

AAA = aortic arch anomalies; Ao/PA = aorta/pulmonary artery; ASO = arterial switch operation; BPV = bicuspid pulmonary valve; LVOTO = left ventricular outflow tract obstruction; NeoAR = neoaortic regurgitation; PAB = pulmonary artery banding; STJ = sinotubular junction; TBA = Taussig-Bing anomaly; VSD = ventricular septal defect.

a Definition of NeoAR (lowest grade included in the pooled analysis).

b Expressed as mean difference.

c OR.

d HR.

### Identification of risk factors associated with neoaortic regurgitation

**Figure 4:**
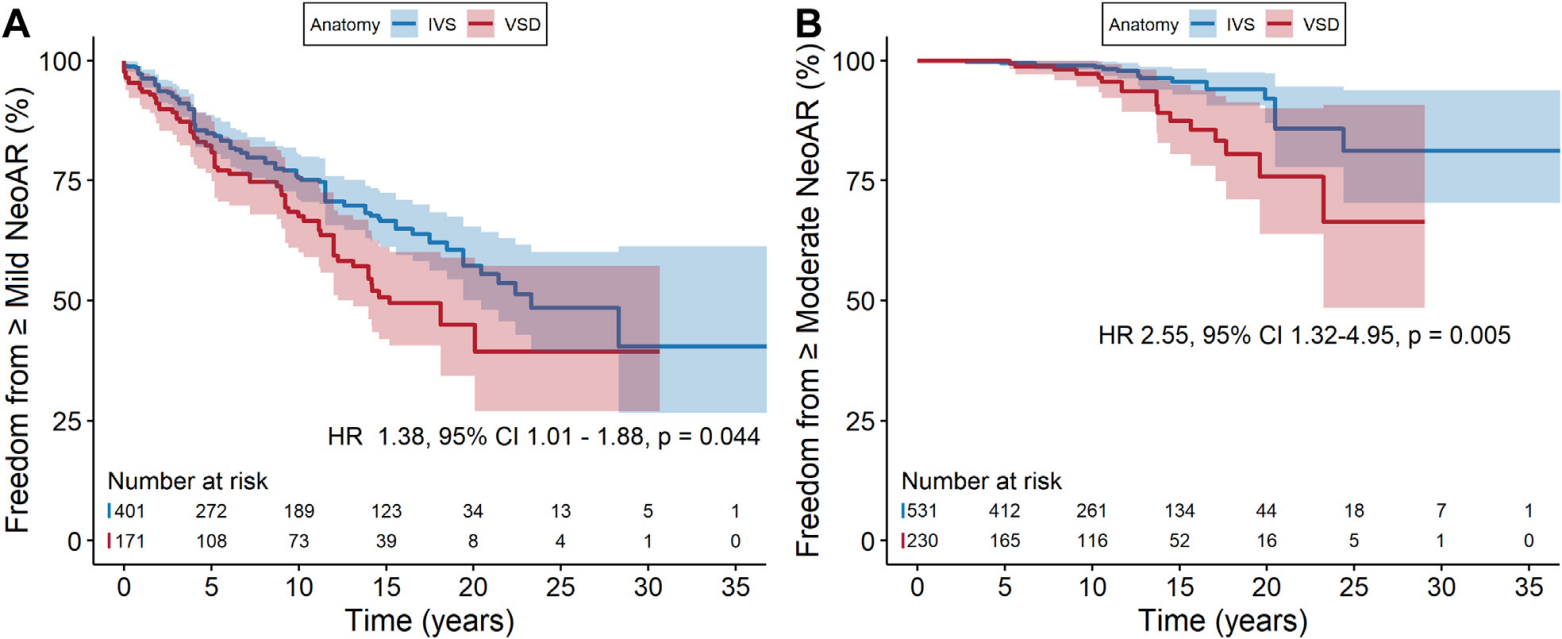
**Cumulative Risk of NeoAR Stratified by Presence of IVS or VSD** Cumulative incidence of (A) ≥mild NeoAR, based on 3 studies^15^^,^^19^^,^^37^ and (B) ≥moderate NeoAR, based on 2 studies.^35^^,^^37^ Presence of a VSD is associated with a higher risk of ≥mild and ≥moderate NeoAR during follow-up, respectively. ASO = arterial switch operation; IVS = intact ventricular septum; NeoAR = neoaortic regurgitation; TGA = transposition of the great arteries; VSD = ventricular septal defect.

Potential risk factors identified on either univariable or multivariable analysis reported from included studies in our literature review are summarized in Supplemental Table 4. To elucidate the association with a ventricular septal defect (VSD), we pooled all studies presenting stratified Kaplan-Meier survival curves. Three studies^15^^,^^19^^,^^37^ compared cumulative incidence of ≥mild NeoAR among patients with an intact ventricular septum with those with a VSD. Two studies^35^^,^^37^ presented cumulative incidence of ≥moderate NeoAR. Patients with an associated VSD had a significantly higher risk of ≥mild and ≥moderate NeoAR during follow-up compared to patients with intact ventricular septum (HR: 1.38 [95% CI: 1.01-1.87], *P* = 0.040; and HR: 2.55 [95% CI: 1.31-4.99], *P* = 0.006, respectively) (Figures 4A and 4B). Results of the meta-analysis comparing risk factors between patients who developed NeoAR after ASO and those who did not are summarized in Table 2. A total of 19 studies compared data on baseline risk factors between patients who developed NeoAR and those who did not (Supplemental Figures 3, 4, 6, and 7). Significant risk factors for developing ≥trace NeoAR included prior pulmonary artery banding (PAB) (OR: 2.83 (95% CI: 1.34-5.96) *P* < 0.001), presence of a VSD (OR: 1.82 [95% CI: 1.07-3.10] *P* = 0.027), aorta/pulmonary artery (Ao/PA) size discrepancy (OR: 2.25 [95% CI: 1.44-3.51], *P* < 0.001), and NeoAR at discharge (OR: 5.64 [95% CI: 3.62-8.79], *P* < 0.001). Significant risk factors for developing ≥moderate NeoAR were prior PAB (HR: 2.56 [95% CI: 1.24-5.29], *P* = 0.011) and presence of a bicuspid pulmonary valve (BPV) (HR: 1.96 [95% CI: 1.01-3.81], *P* = 0.047).

## Discussion

In our systematic review, we analyzed 30 retrospective observational studies on ASO involving 6,169 patients across 13 countries. We demonstrate that in a large group of patients with TGA followed for 30 years after ASO, 32.5% of patients remained free from ≥ mild NeoAR, with 78.6% of patients remaining free from ≥moderate NeoAR (Central Illustration). Additionally, our meta-analysis reveals associations between larger neoaortic Z-scores and the occurrence of NeoAR and identifies 5 key risk factors linked to the development of NeoAR, including the presence of a VSD, BPV, prior PAB, Ao/PA size discrepancy, and the occurrence of NeoAR at discharge. Despite the fact that NeoAR and NeoARD may not constitute a significant clinical problem in many patients, as shown by the high freedom from reoperation on the neoaortic root or neoaortic valve during follow-up,^38^ significant NeoAR does ultimately occur in an important minority of patients. Given that the phenomena of NeoAR and NeoARD are progressive, it is crucial to acknowledge that within the long-term survivors post-ASO, the impact of NeoAR and NeoARD are also anticipated to increase. This emphasizes the clinical significance of our work, as monitoring the progression of NeoAR and its associated risk factors offers critical insights into those at risk and aids the continued cardiovascular care pathway as patients age.

**Central Illustration:**
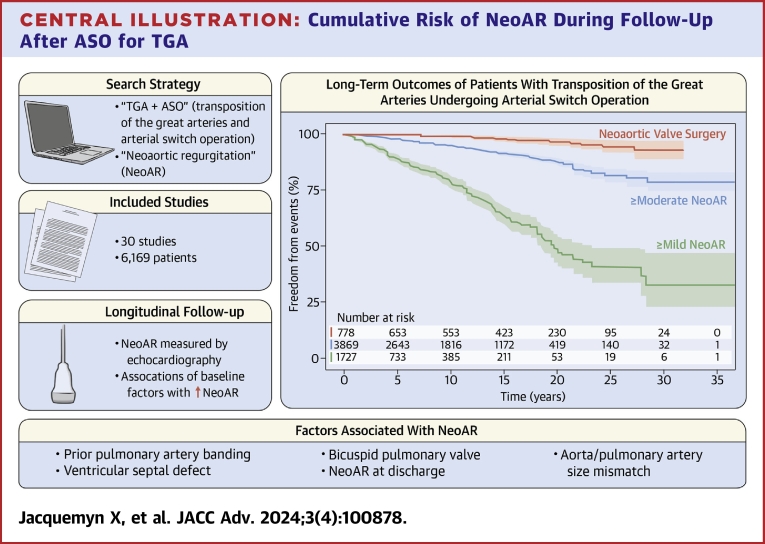
**Cumulative Risk of NeoAR During Follow-Up After ASO for TGA** ASO = arterial switch operation; NeoAR = neoaortic regurgitation; TGA = transposition of the great arteries.

The recognition of both internal and external risk factors suggests that multiple mechanisms are likely responsible for the production of NeoAR and NeoARD after ASO. Internal risk factors include some well-documented histological differences between patients with TGA and a normal healthy population. The pulmonary valve has thinner leaflets and a diminished amount of collagen and elastic fibers in comparison to the native aortic valve, and the arterial roots show differing distribution of collagen, which is diminished in the pulmonary artery.^39^ Studies of TGA have shown that both arterial roots and the neoaortic valve show less extensive anchorage and embedding in the myocardium, and that the neoaortic root and pulmonary valve annulus are already larger prior to ASO in comparison to healthy neonates. External risk factors include the altered geometry of the neoaortic root following the ASO with Lecompte procedure, leading to increased helical flow patterns that cause changes in aortic wall shear forces and thus progressive dilatation.^12^ Other proposed external contributors to neoaortic dilatation include implantation of the coronary arteries inducing a widening of the neoaortic root, disruption of the vasa vasorum around the neoaorta, male sex (potentially explained by larger baseline aortic root dimensions and hormonal differences), and being overweight.^14^^,^^17^^,^^35^^,^^37^

We identified 5 significant risk factors associated with NeoAR, including prior PAB, a VSD, BPV, Ao/PA size discrepancy, NeoAR at hospital discharge, and demonstrate an association between greater neoaortic root dimensions and concurrent NeoAR. The exact pathophysiological mechanisms through which these risk factors cause NeoARD and NeoAR remain unclear. Potentially, a VSD can cause neoaortic dilatation due to increased pulmonary valve blood flow in fetal life, caused by increased oxygen saturation and decreased resistance in the pulmonary vasculature, resulting in larger dimensions even before ASO,^22^ or from pulmonary artery pressure elevation inducing changes in muscle fiber patterns.^40^ None of these risk factors are easily modifiable, as the choice for PAB is frequently based on significant comorbidities or late diagnosis, and Ao/PA size discrepancy and presence of VSD or BPV are inherent structural risk factors. A PAB is often done as a temporary measure for left ventricular (LV) “training,” where the LV is deemed unfit to support the systemic pressures. The mechanism through which Ao/PA size discrepancy plays a role in NeoAR and NeoARD is suggested to be related to the altered geometry of the roots influencing fluid dynamics, as larger wall shear stress (WSS) magnitudes are detected in patients with relatively small mid-ascending aortic diameter when compared with the neoaortic root.^41^ Levels of WSS play several essential roles in functions of endothelial cells and have been demonstrated to promote initiation and development of various vascular pathologies, among which aortic aneurysms.^42^ Furthermore, it has been demonstrated that, after ASO, the flow hemodynamics are significantly asymmetric between different regions of the neoaortic root and ascending aorta, which may explain the variations in regional vessel wall remodeling along the aorta and, additionally, why some regions are more prone to dilatation.^43^ Despite our findings indicating increased risk with the aforementioned factors, the independent effects of PAB or Ao/PA size discrepancy are hard to estimate since results may be confounded by the presence of a VSD (eg, the hemodynamic effect from a VSD might contribute to create a size difference between the aorta and pulmonary artery). Then, a BPV introduces hemodynamic differences with both increased tensile and WSS and more turbulent blood flow, resulting in an uneven force distribution on the convex wall of the ascending aorta.^33^

A proposed preventative measure for NeoARD is pulmonary artery reduction during the initial ASO for those with severe forms of Ao/PA size discrepancy (>2:1 ratio of PA to Ao).^44^ Additionally, if reliable means could be developed to prevent neoaortic dilation, it could also favorably impact NeoAR by reducing intercommisural distance and promoting more effective leaflet coaptation. Patients with progressive aortic dilatation may develop problems related to external compression of main and branch PAs, resulting in a decreased pulmonary blood flow and PA stenosis.^45^ Compression, kinking, or stretching of the coronaries can occur, with late coronary stenosis or occlusion as a result.^46^

NeoAR is expected to become increasingly important as the ASO population ages, and interventions to treat both NeoAR and NeoARD will become more commonplace. In the original studies, surgery was indicated for various conditions, primarily involving significant NeoAR with or without significant LV dilatation in the majority of cases. Additional indications included progressive NeoARD, significant NeoAR in the presence of concomitant subaortic tunnel stenosis, significant NeoAR with both LV dilatation and impaired LV function, and a singular case involving refractory cardiac failure accompanied by LV dilatation (Supplemental Table 3). However, the observed discordance between the prevalence of significant NeoAR and the proportion of patients undergoing NeoAV surgery, as highlighted in Supplemental Table 3, raises important questions regarding the factors contributing to this discrepancy. Specifically, there was a discrepancy of 269 patients with ≥moderate NeoAR (5.9% of total population, from 18 studies), but only 91 (1.7%) underwent surgery. We suspect this discrepancy may reflect differences in institutional practices and intrinsic differences in specific measures of NeoAR, NeoARD, and ventricular dimensions. Another factor that may attribute to this difference is the era effect, since our study includes reports published over 2 decades. For NeoAR with symptoms and/or progressive dilatation of the left ventricle, our recommended indications for surgery align with the guidelines.^47^ However, international guidelines for surgical intervention on NeoARD are primarily based on data from other forms of degenerative aortic disease or bicuspid aortic valves. Yet, our understanding of the natural progression of aneurysms after arterial ASO is still limited, as there have been no published reports of aortic catastrophe. Considering the growing evidence of the progressive nature of NeoARD in this relatively young population, early surgical intervention may be justified. In contrast, emphasizing the importance of noninvasive lifestyle interventions, particularly for overweight individuals who often exhibit higher blood pressure and larger neoaortic diameters,^17^ we advocate for promoting physical activity. Physical activity has demonstrated benefits for fitness, psychological well-being, and overall heart health, positioning it as a pivotal element in comprehensive congenital heart disease management.^48^

Lastly, the predictive power of known risk factors for NeoAR and NeoARD is imperfect, so all patients should be imaged regularly, irrespective of the presence or absence of risk factors. Future research should focus on the development of effective risk assessment strategies and preventive approaches to mitigate adverse sequelae following ASO. Additionally, more follow-up studies measuring neoaortic growth further into adulthood are necessary.

### Study Limitations

A number of limitations should be considered when interpreting the present study. First, this meta-analysis summarizes data obtained primarily from heterogeneous retrospective observational studies. Second, we may have lacked statistical power to identify some previously proposed risk factors in individual studies, as some risk factors simply could not be analyzed using these methods and the data currently available, explaining the discrepancy between the amount of potential risk factors (Supplemental Table 4) and those included in our analysis (Table 2). Then, results from the fixed-effect analysis need to be interpreted with caution, since plausible violation can occur upon combination of results obtained from small studies, where statistical imprecision in the study’s estimated standard errors are considerable. In addition, it has been demonstrated that inconsistencies in qualitative echo grading of aortic regurgitation are widespread and that echo is less reliable and often overestimates severity when compared to cardiac magnetic resonance imaging.^49^ Nevertheless, our study used all available echocardiographic data to summarize the incidence of and risk factors for NeoAR after ASO and may therefore represent more generalizable reference values than those reported by individual centers.

## Conclusions

The currently available literature demonstrates that ASO for TGA is associated with progressive NeoAR. This synthesis of published observations estimates that approximately 67.5% of patients develop ≥mild NeoAR after 30 years post-ASO. For most patients, however, the overall performance of the neoaortic valve remains reasonably good, as ≥moderate NeoAR occurs in only 21.4% of patients at 30 years after ASO. We identified clinical risk factors, most of which are related to neoaortic root deformities, associated with the development of NeoAR during follow-up after ASO. The need for neoaortic root and valve reinterventions remains minimal during the initial 10 to 15 years following ASO but exhibits a gradual upward trend with progression towards significant NeoAR and NeoARD. Future research should focus on refining our understanding of risk factors and the mechanisms by which they promote neoaortic valve and root disease so that effective preventative strategies can be implemented.

PERSPECTIVES **COMPETENCY IN MEDICAL KNOWLEDGE:** Development of neoaortic regurgitation and neoaortic dilation in patients who underwent the arterial switch operation for transposition of the great arteries is progressive and is associated with several risk factors.

**COMPETENCY IN PATIENT CARE:** Occurrence of neoaortic regurgitation and neoaortic dilation increases in an aging arterial switch operation population, and as a result, the evaluation and management of patients should be considerate of these long-term outcomes.

**TRANSLATIONAL OUTLOOK:** A better understanding of the clinical importance of neoaortic regurgitation and neoaortic dilation is needed.

## Funding support and author disclosures

This research did not receive any specific grant from funding agencies in the public, commercial, or not-for-profit sectors. The authors have reported that they have no relationships relevant to the contents of this paper to disclose.
