# Supplementary material for: Neoaortic Regurgitation Detected by Echocardiography After Arterial Switch Operation: A Systematic Review and Meta-Analysis
Source: JACC Adv. 2024 Mar 7;3(4):100878. doi: 10.1016/j.jacadv.2024.100878 (PMC11198711; doi:10.1016/j.jacadv.2024.100878)
Supplement: Supplemental data [file mmc1.docx]

**SUPPLEMENTAL APPENDIX**

**Supplemental Methods**

Additional exclusion criteria

Studies were excluded if the population comprised only of patients with complex TGA lesions. For instance, studies reporting only on TGA patients with associated left ventricular outflow obstructions (LVOTO), aortic arch anomalies (AAA), bicuspid pulmonary valve (BPV) or multiple ventricular septal defects (VSD) were excluded to study a representative TGA population and prevent significant study population heterogeneity. Additionally, language was restricted to English. Reviews, letters to the editor, case reports and case series were excluded.

Study Selection

The following steps were undertaken for study selection: 1) identification of records through database search; 2) removal of duplicates; 3) screening and selection of abstracts; 4) assessment for eligibility using full-text articles; and 5) final inclusion in study.

Statistical Analysis

Data extraction of from published Kaplan-Meier graphs of the included studies was performed using a two-step approach. In the first stage, Kaplan-Meier plots were digitized to raw data coordinates using Web Plot Digitizer software. In the second step, individual patient data was reconstructed from the raw data coordinates obtained from the digitized Kaplan-Meier graphs and the respective numbers at risk at given timepoints using the R package “IPDfromKM” (version 0.1.10). To confirm the quality of the reconstructed individual patient data, we thoroughly checked the consistency with the reported Kaplan-Meier plots provided in the original publications and evaluated difference between estimated and read−in suvival probabilities both visually and using accuracy measures provided by the “IPDfromKM” package (root mean square error, mean absolute error and max absolute error).

Cox regression models were for used analysis of TGA subtypes. Using a Cox frailty model, the subtypes were included in the model as a fixed effect. A robust variance estimator was used in the model. Between-study heterogeneity was assessed by the inclusion of a γ frailty term, where individual studies modelled as a random effect using random intercepts.

The proportionality of the hazards of each Cox model was assessed with the Grambsch-Therneau test and diagnostic plots based on Schoenfeld residuals. The assumption of proportionality of the hazards was met in both models (P = 0.888 and P = 0.795, respectively).


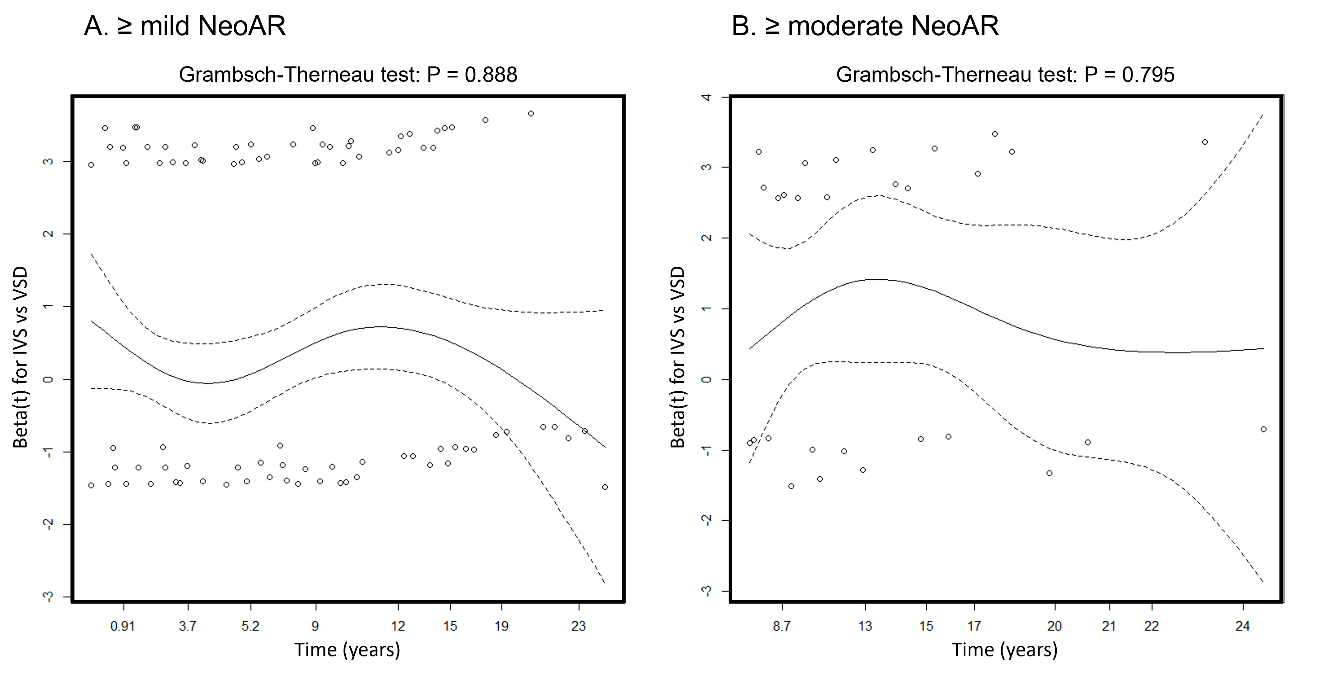


In addition, to identify risk factors of NeoAR during follow-up, odds ratios (OR), hazard ratios (HR) and mean differences (MD) from individual studies were pooled using random-effects models. Baseline variables reported in 3 or more studies were considered for pooling. I², describing the percentage of total variation across studies that is attributable to heterogeneity rather than chance, was calculated to assess the degree of statistical heterogeneity, and its accompanying p value was obtained using the chi-square test of the Cochran Q heterogeneity statistic. **Supplemental Figure 1. Risk of bias summary – ROBINS-I tool traffic lights (A) and summary plot (B).**


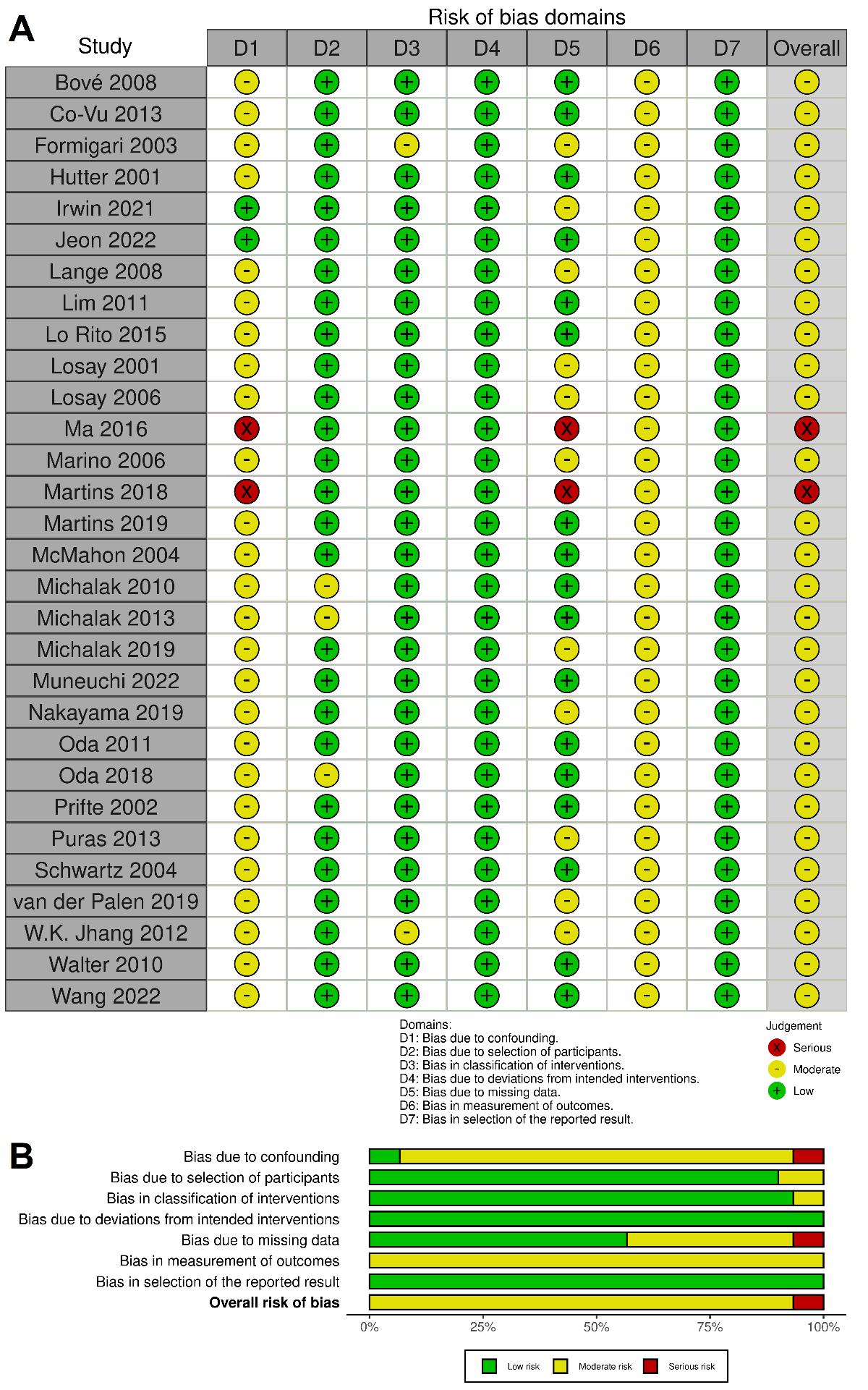


**Supplemental Figure 2. Forest plots summarizing the meta-analysis of neoaortic root Z-scores between patients with and without NeoAR during follow-up.**


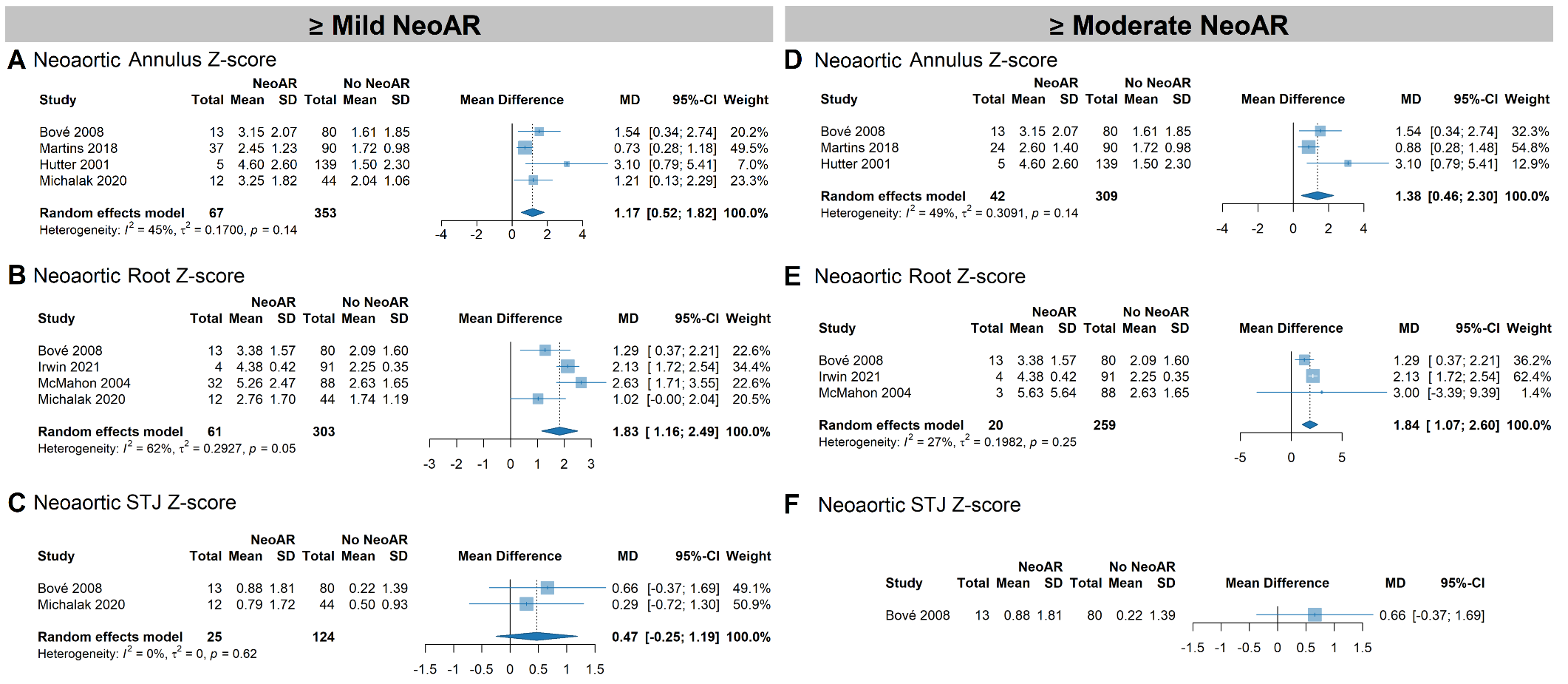


LEGEND: Differences in neoaortic root Z-scores at (A) aortic annulus (B) aortic root and (C) aortic sinotubular junction (STJ) Z-scores between patients with ≥ mild NeoAR and those without NeoAR and neoaortic root Z-scores at (D) aortic annulus (E) aortic sinus and (F) aortic STJ Z-scores between patients with ≥ moderate NeoAR and those without NeoAR.

**Supplemental Figure 3. Forest plots summarizing the risk factors associated with the development of any NeoAR (≥ trace) during follow up.**


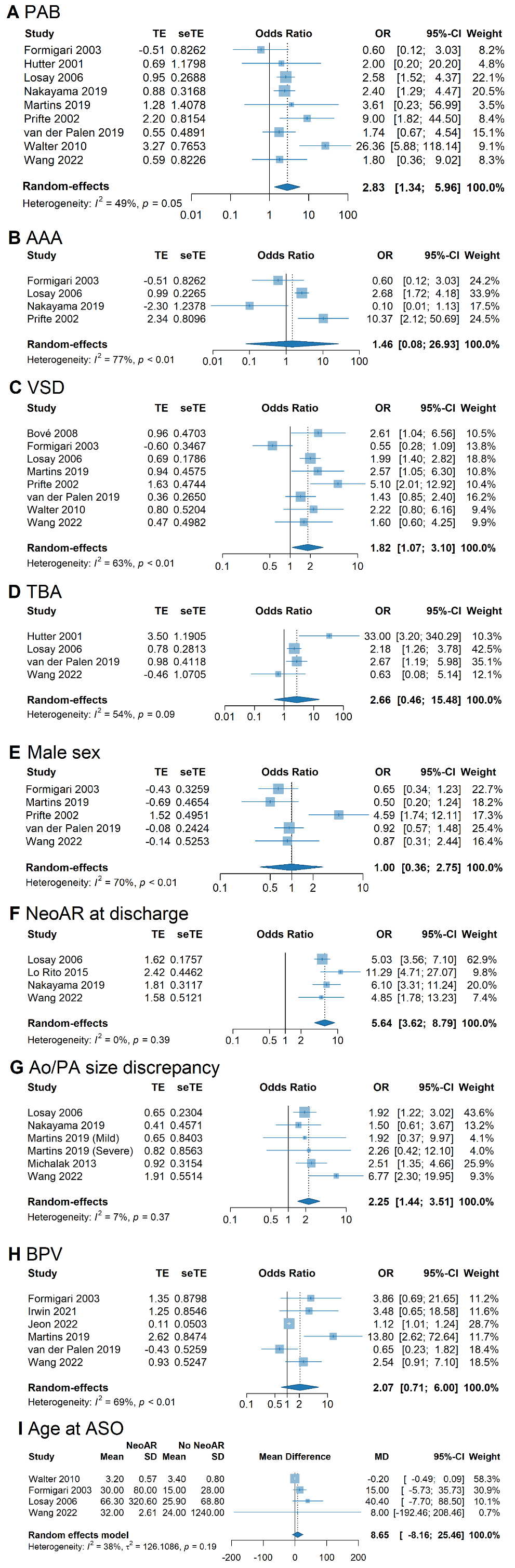


LEGEND: Included studies assessed risk of ≥ trace NeoAR during follow-up. ASO, arterial switch operation; Ao, aorta; CI, confidence interval; HR, hazard ratio; MD, mean difference; NeoAR, neoaortic regurgitation; OR, odds ratio; PA, pulmonary artery; PAB, pulmonary artery binding; TBA, Taussig-Bing anomaly; VSD, ventricular septal defect.

**Supplemental Figure 3. Forest plots summarizing the risk factors associated with the development of any NeoAR (≥ trace) during follow up. (continued).**


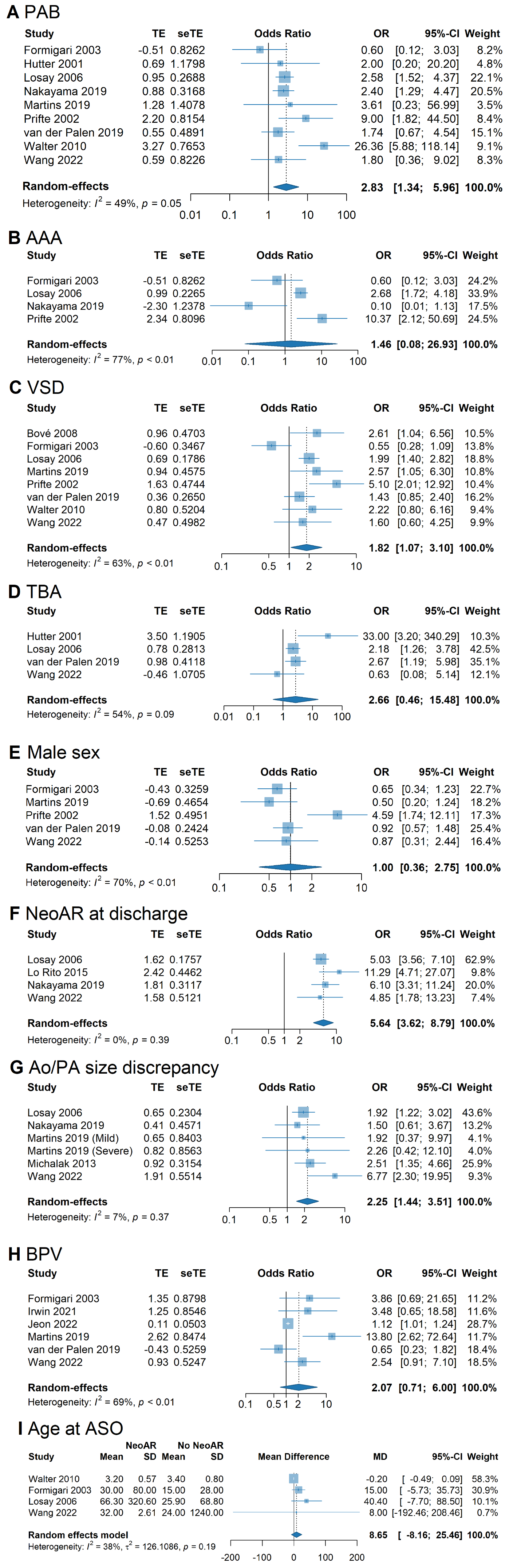


LEGEND: Included studies assessed risk of ≥ trace NeoAR during follow-up. ASO, arterial switch operation; Ao, aorta; CI, confidence interval; HR, hazard ratio; MD, mean difference; NeoAR, neoaortic regurgitation; OR, odds ratio; PA, pulmonary artery; PAB, pulmonary artery binding; TBA, Taussig-Bing anomaly; VSD, ventricular septal defect.

**Supplemental Figure 4. Forest plots summarizing the risk factors, from studies reporting hazard ratios, associated with the development of NeoAR (≥ moderate) during follow up.**


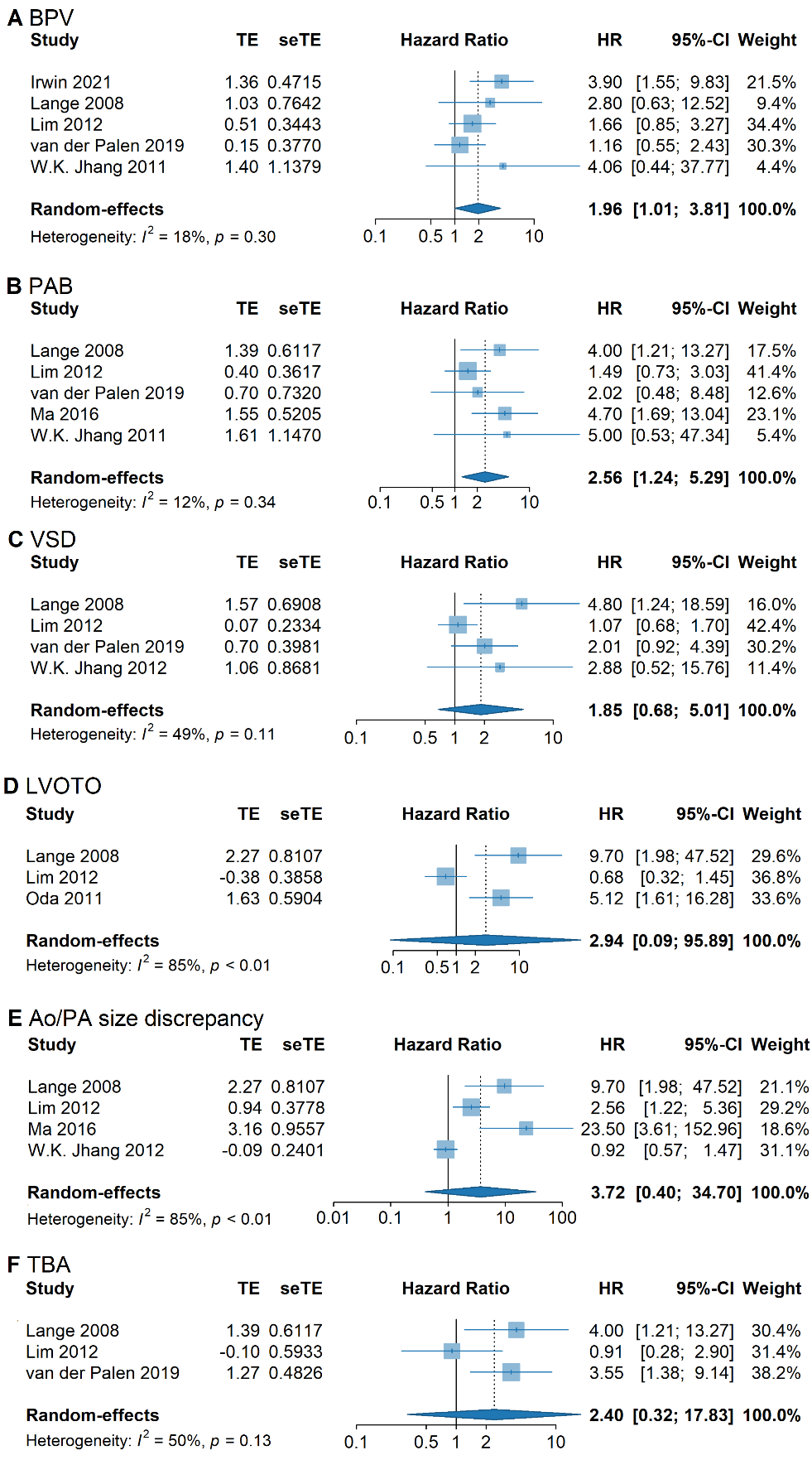


LEGEND: All included studies assessed risk of ≥ moderate NeoAR during follow-up. CI, confidence interval; HR, hazard ratio; LVOTO, left ventricular outflow tract obstruction; PA, pulmonary artery; PAB, pulmonary artery binding; TBA, Taussig-Bing anomaly; VSD, ventricular septal defect.

**Supplemental Figure 5. Sensitivity Fixed Effects Analysis - Forest plots summarizing the meta-analysis of neoaortic root Z-scores between patients with and without NeoAR during follow-up.**


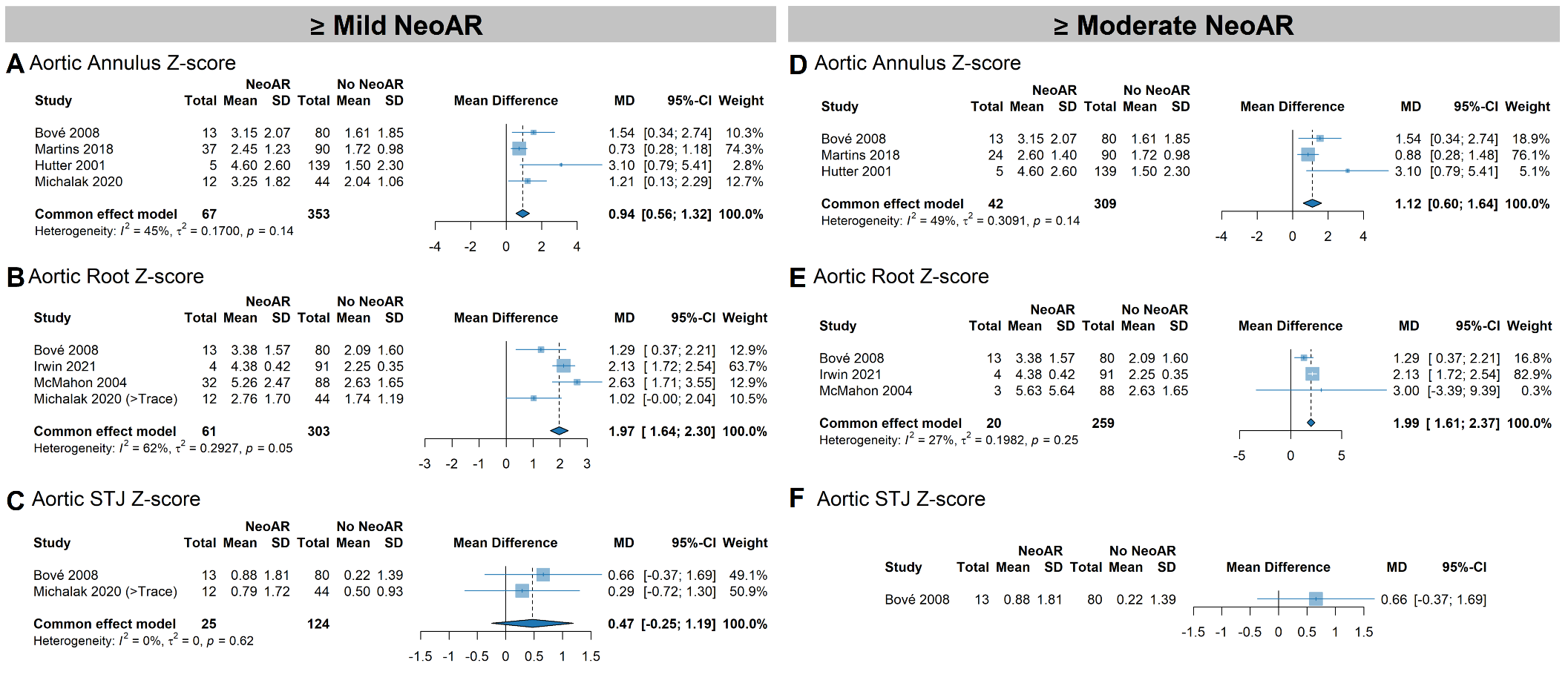


LEGEND: Differences in neoaortic root Z-scores at (A) aortic annulus (B) aortic root and (C) aortic sinotubular junction (STJ) Z-scores between patients with ≥ mild NeoAR and those without NeoAR and neoaortic root Z-scores at (D) aortic annulus (E) aortic sinus and (F) aortic STJ Z-scores between patients with ≥ moderate NeoAR and those without NeoAR.

**Supplemental Figure 6. Sensitivity Fixed Effects Analysis - Forest plots summarizing the risk factors associated with the development of any NeoAR (≥ trace) during follow up.**


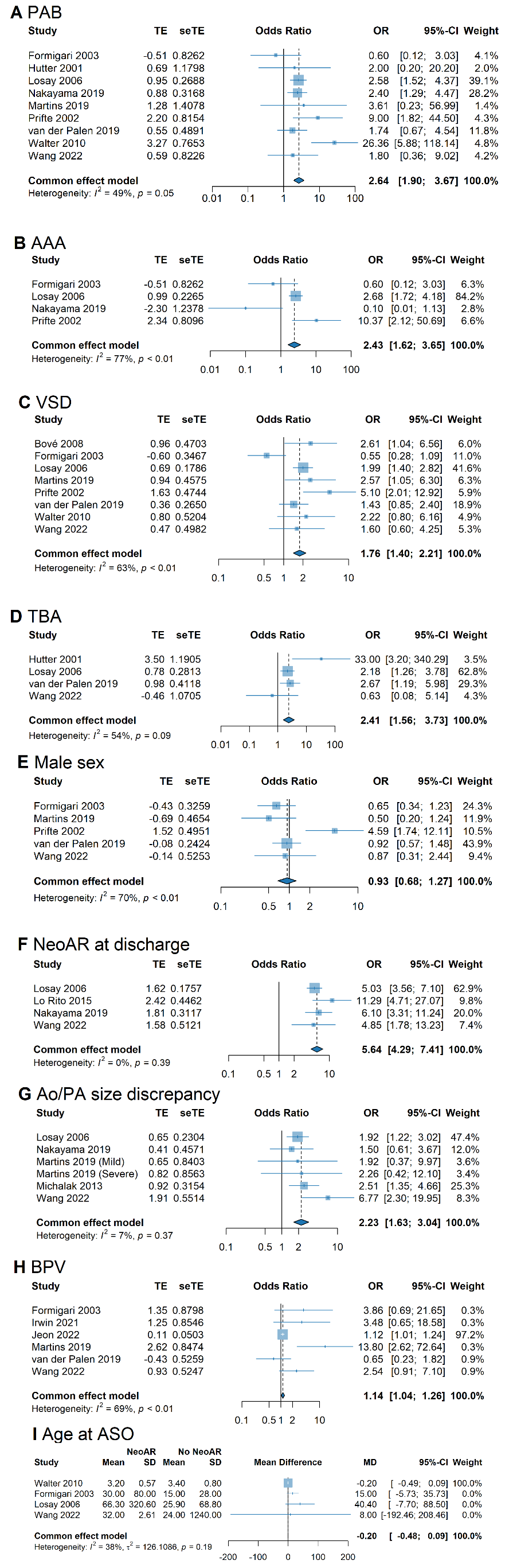


LEGEND: Included studies assessed risk of ≥ trace NeoAR during follow-up. ASO, arterial switch operation; Ao, aorta; CI, confidence interval; HR, hazard ratio; MD, mean difference; NeoAR, neoaortic regurgitation; OR, odds ratio; PA, pulmonary artery; PAB, pulmonary artery binding; TBA, Taussig-Bing anomaly; VSD, ventricular septal defect.

**Supplemental Figure 6. Sensitivity Fixed Effects Analysis - Forest plots summarizing the risk factors associated with the development of any NeoAR (≥ trace) during follow up. (continued).**


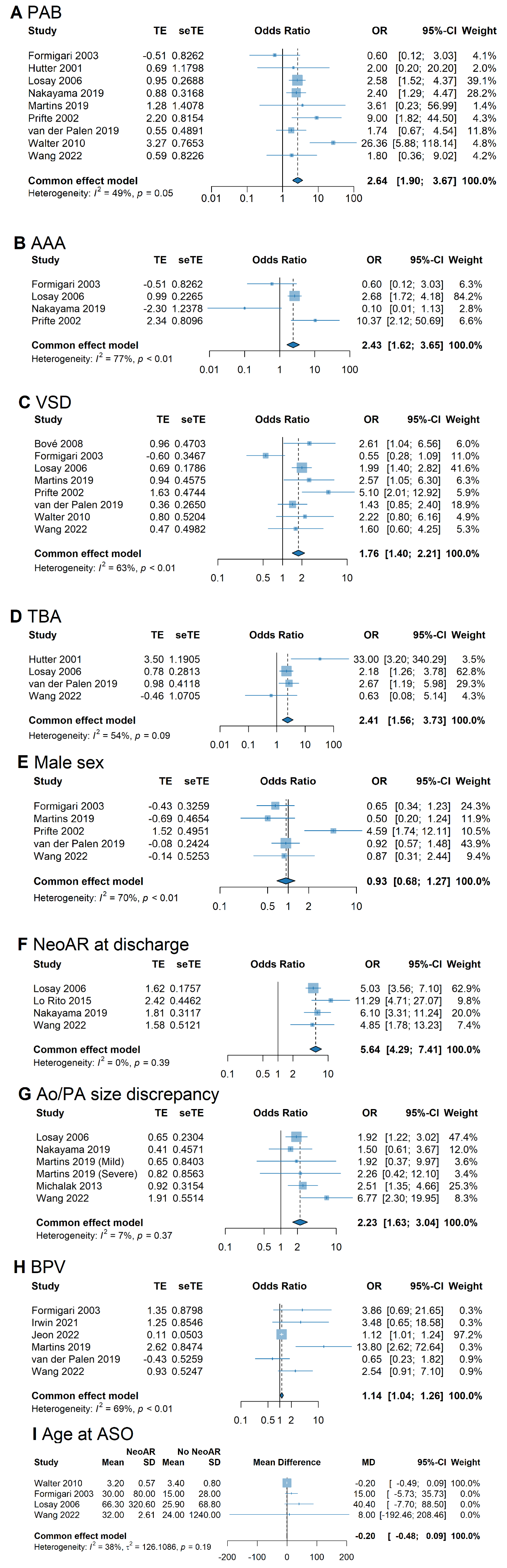


LEGEND: Included studies assessed risk of ≥ trace NeoAR during follow-up. ASO, arterial switch operation; Ao, aorta; CI, confidence interval; HR, hazard ratio; MD, mean difference; NeoAR, neoaortic regurgitation; OR, odds ratio; PA, pulmonary artery; PAB, pulmonary artery binding; TBA, Taussig-Bing anomaly; VSD, ventricular septal defect.

**Supplemental Figure 7. Sensitivity Fixed Effects Analysis - Forest plots summarizing the risk factors, from studies reporting hazard ratios, associated with the development of NeoAR (≥ moderate) during follow up.**


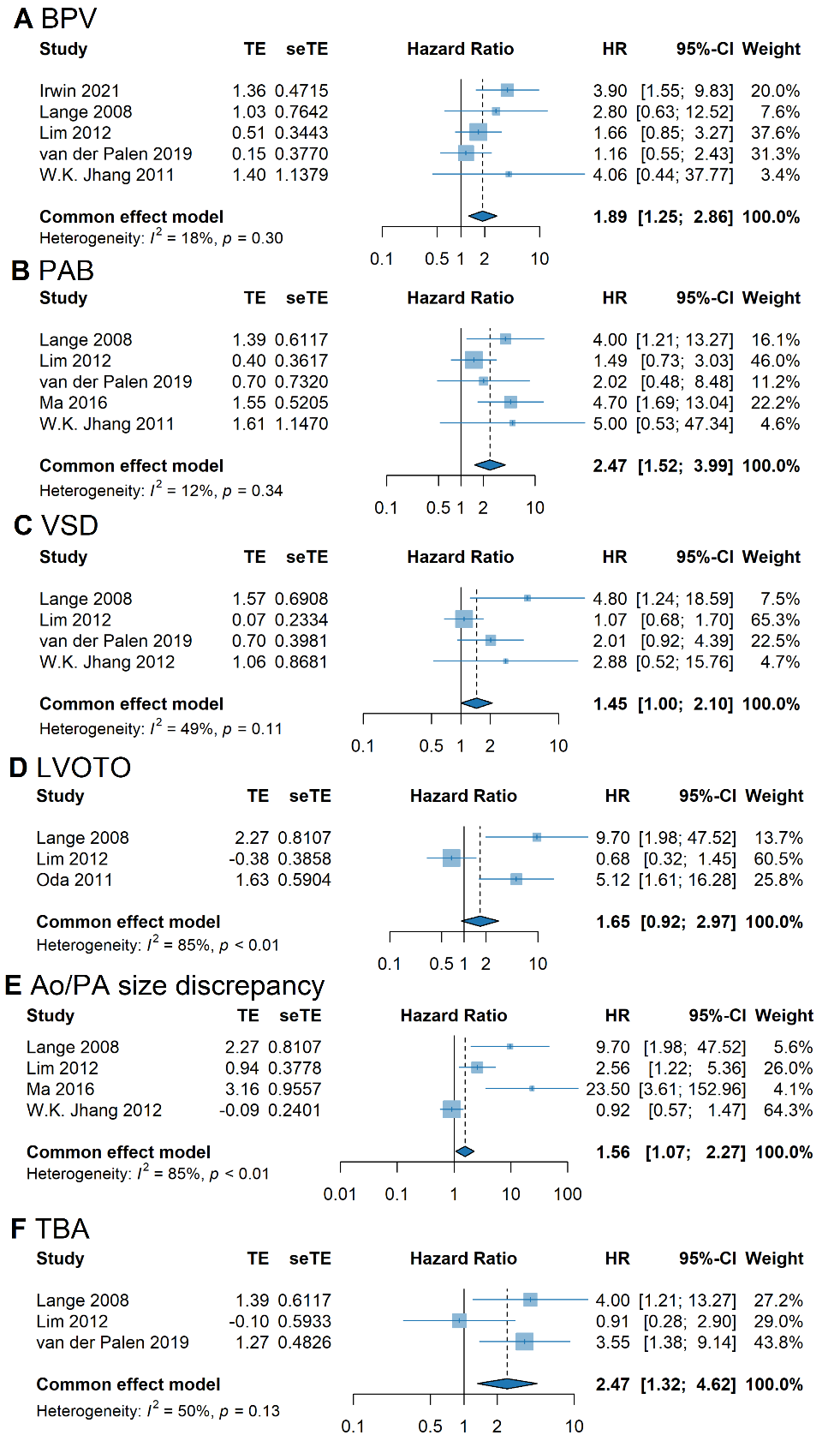


LEGEND: All included studies assessed risk of ≥ moderate NeoAR during follow-up. CI, confidence interval; HR, hazard ratio; LVOTO, left ventricular outflow tract obstruction; PA, pulmonary artery; PAB, pulmonary artery binding; TBA, Taussig-Bing anomaly; VSD, ventricular septal defect

**Supplemental Table 1. Search strategy**

| Database | Search String |
| --- | --- |
| PubMed/MEDLINE  (n = 135) | ("Transposition of Great Vessels"[Mesh] OR "Transposition of the Great Arteries, Dextro-Looped 1" [Supplementary Concept] OR "Transposition of the Great Arteries” OR “d-tga” OR “dtga” OR “Ventriculo-Arterial Discordance” OR “Corrected Transposition of the Great Arteries”) AND ("Arterial Switch Operation"[Mesh] OR “Jatene” OR “Arterial Switch” OR “Lecompte”) AND ("Aortic Valve Insufficiency"[Mesh] OR “Neoarta” OR “Neoartic Regurgitation” OR “Neoaortic Root” OR “Neoaortic Valve Regurgitation” OR “Neoaortic Root Dilatation” OR “Aortic Aneurysm” OR “Neoaortic Valve Injury”) |
| Embase  (n = 328) | (‘Transposition of Great Vessels’ OR ‘Transposition of the Great Arteries’ OR ‘d-tga’ OR ‘dtga’ OR ‘Ventriculo-Arterial Discordance’ OR ‘Corrected Transposition of the Great Arteries’) AND (‘Arterial Switch Operation’ OR ‘Jatene’ OR ‘Arterial Switch’ OR ‘Lecompte’) AND (‘Aortic Valve Insufficiency’ OR ‘Neoarta’ OR ‘Neoartic Regurgitation’ OR ‘Neoaortic Root’ OR ‘Neoaortic Valve Regurgitation’ OR ‘Neoaortic Root Dilatation’ OR ‘Aortic Aneurysm’ OR ‘Neoaortic Valve Injury’) |
| Scopus  (n = 131) | (TITLE-ABS-KEY(“Transposition of Great Vessels”) OR TITLE-ABS-KEY(“Transposition of the Great Arteries”) OR TITLE-ABS-KEY(“Ventriculo-Arterial Discordance”) OR TITLE-ABS-KEY(d-tga)) AND (TITLE-ABS-KEY(“Arterial Switch Operation”) OR TITLE-ABS-KEY(Jatene) OR TITLE-ABS-KEY(“Arterial Switch”) OR TITLE-ABS-KEY(Lecompte)) AND (TITLE-ABS-KEY(“Aortic Valve Insufficiency”) OR TITLEABS-KEY(Neoarta) OR TITLE-ABS-KEY(“Neoartic Regurgitation”) OR TITLE-ABS-KEY(“Neoaortic Root”) OR TITLE-ABS-KEY(“Neoaortic Valve Regurgitation”) OR TITLE-ABS-KEY(“Neoaortic Root Dilatation”) OR TITLE-ABS-KEY(“Neoaortic Valve Injury”)) |

**Supplemental Table 2. Method of Assessment of NeoAR in individual studies**

| Study | NeoAR,  n (%) | Grading of NeoAR | Method for assessment of NeoAR |
| --- | --- | --- | --- |
| Bové *et al.*^1^ | 13 (14) | NA | “Flow velocity acceleration and assessment of aortic regurgitation by using the quantitative grading.” |
| Co-Vu *et al.*^2^ | 17 (14) | Semiquantitative | Width of the color Doppler: 1-4 mm (Trivial-Mild), 4-6 mm (Moderate), >6 mm (Severe); Moderate and severe regurgitation were verified by left ventricular dilatation and presence of diastolic flow reversal in the aortic arch or descending aorta. |
| Formigari *et al.*^3^ | 61 (35) | Quantitative | Trivial if a regurgitant jet was identified in the absence of diastolic flow reversal on pulsed wave Doppler interrogation of the ascending aorta. Hemodynamically significant in presence of diastolic flow reversal.^1^ |
| *Hutter et al.*^4^ | 5 (3.5) | NA | NA |
| Irwin *et al.*^5^ | 4  (4.2) | NA | “Extracted as descriptive data from echocardiography report” |
| Jeon *et al.*^6^ | NA | NA | “Doppler echocardiographic findings following the American Society of Echocardiography guideline” |
| Lange *et al.*^7^ | 41 (8.7) | Semiquantitative | Quantification was based on the following parameters: trivial aortic insufficiency (grade I): jet length less than one third of left ventricle (LV) length, LV normal sized. Mild aortic insufficiency (grade II): jet length one half of LV length, vena contracta less than 10% of aortic valve annulus diameter, no diastolic regurgitation from descending aorta. Moderate aortic insufficiency (grade III): jet length to the apex of LV, LV diameter normal or slightly enlarged, vena contracta less than 10—20% of aortic valve annulus, early to mid-diastolic regurgitation from descending aorta. Severe aortic insufficiency (grade IV): broad jet to the LV apex, LV diameter dilated, function hypercontractile or diminished, vena contracta >20% of aortic valve annulus, holodiastolic regurgitation from descending aorta. |
| Lim *et al.*^8^ | 78  (38.0) | Quantitative | Ratio of the width of the regurgitant jet to the diameter of the left ventricular outflow tract. |
| Lo Rito *et al.*^9^ | 97 (45.8) | NA | NA |
| Losay *et al.*^10^ | 165  (15.5) | Quantitative | Ratio of the width of the regurgitant jet to the diameter of the left ventricular outflow tract. |
| Losay *et al.*^11^ | 172 (14.9) | Quantitative | Ratio of the width of the regurgitant jet to the diameter of the left ventricular outflow tract. |
| Ma *et al.*^12^ | 56 (10.4) | Quantitative | Ratio of the width of the regurgitant jet to the diameter of the left ventricular outflow tract. |
| Marino *et al.*^13^ | 69 (84.1) | Semiquantitative | Width of the color Doppler: 1-4 mm (Trivial-Mild), 4-6 mm (Moderate), >6 mm (Severe); Moderate and severe regurgitation were verified by left ventricular dilatation and presence of diastolic flow reversal in the aortic arch or descending aorta. |
| Martins *et al.*^14^ | 37 (29.1) | Quantitative | Ratio of the width of the regurgitant jet to the diameter of the left ventricular outflow tract, <0.25 (Mild), 0.25-0.50 (Moderate), >0.50 (Severe). In the presence of holodiastolic flow reversal in the descending aorta, regurgitation was considered moderate or severe. |
| Martins *et al.*^15^ | 35 (22) | NA | "Classified based on quantitative and qualitative data" |
| McMahon *et al.*^16^ | 32 (27) | Semiquantitative | Width of the color Doppler: 0 mm (None),>0.5 mm (Trivial), 1-3 mm (Mild), 3-5 mm (Moderate), >5 mm (Severe). |
| Michalak *et al.*^17^ | 75 (47) | Semiquantitative | Ratio of the diameter and area of the regurgitant jet to aortic annulus diameter and area; presence of diastolic reversal flow in the descending aorta; presence of LV enlargement. |
| Michalak *et al.*^18^ | 85 (49) | Semiquantitative | Ratio of the diameter and area of the regurgitant jet to aortic annulus diameter and area; presence of diastolic reversal flow in the descending aorta; presence of LV enlargement. |
| Michalak *et al.*^19^ | 30 (53.6) | Semiquantitative | Ratio of the width of the regurgitant jet to the diameter of the left ventricular outflow tract; presence of diastolic reversal flow in the descending and abdominal aorta; the pressure half time and LV end-diastolic diameter and volume. |
| Muneuchi *et al.*^20^ | 8 (17.8) | NA | NA |
| Nakayama *et al.*^21^ | 41 (8.6) | Quantitative | Ratio of the width of the regurgitant jet to the diameter of the left ventricular outflow tract. |
| Oda *et al.*^22^ | 29  (7.5) | Quantitative | Ratio of the width of the regurgitant jet to the diameter of the left ventricular outflow tract, <0.25 (Mild), 0.25-0.64 (Moderate), >0.65 (Severe). |
| Oda *et al.*^23^ | 21 (14.5) | NA | NA |
| Prifti *et al.*^24^ | 10 (7.5) | NA | NA |
| Puras *et al.*^25^ | 43  (28) | NA | NA |
| Schwartz *et al.*^26^ | 17 (5.1) | NA | "Assessment of AR was taken from the original echocardiographic reports" |
| van der Palen *et al.*^27^ | 33 (9.6) | Quantitative | Width of the color Doppler: 0-1 mm (Trivial), 1-4 mm (Mild), 5-6 mm (Moderate), >6 mm (Severe). |
| W.K. Jhang *et al.*^28^ | 6 (2.5) | NA | “Colour Doppler imaging and quantitatively graded” |
| Walter *et al.*^29^ | 17 (5.2) | Quantitative | Ratio of the width of the regurgitant jet to the diameter of the left ventricular outflow tract. |
| Wang *et al.*^30^ | 19 (11.5) | Quantitative | Ratio of the width of the regurgitant jet to the diameter of the left ventricular outflow tract. |

LEGEND: NeoAR, neoaortic regurgitation.

**Supplemental Table 3. Summary of NeoAVS in included studies.**

| Study | Year | Sample  size (n) | Follow-up  (years) | NeoAR*, n (%) | NeoAVS, n (%) | Intervention | Indication for intervention |
| --- | --- | --- | --- | --- | --- | --- | --- |
| Bové *et al.*^1^ | 2008 | 93 | 4.8±3.9 | 1 (1.1) | 1 (1.1) | AV- and root plasty with modified Konno (1) | Concomitant subaortic tunnel stenosis and significant AR |
| Co-Vu *et al.*^2^ | 2013 | 124 | 7.2  (1-23) | 17 (14.0) | 0 (0) | None | - |
| Formigari *et al.*^3^ | 2003 | 173 | 8.2  (0.6-11.2) | 2 (1.2) | 2 (1.2) | AV plasty (1)†, Mechanical AVR (1) | mAVR: Progressive regurgitant fraction |
| Irwin *et al.*^5^ | 2021 | 278 | 11.3  (0.02-30.3) | 18 (6.4) | 8 (4.0) | AV repair (2), AV repair and root reduction (2), ARR (2), AVR (2) | NA |
| Lange *et al.*^7^ | 2008 | 479 | 9.3  (0-22.6) | 7 (1.5) | 11 (2.3) | Isolated mechanical AVR (4) and AVR with additional procedures (7) | NA |
| Lo Rito *et al.*^9^*†* | 2015 | 362 | 16  [12-18.2] | 27 (8.7) | 5 (1.4) | AVR (3), Bentall procedure (1), AV repair (1) | Refractory cardiac failure and LV dilatation (1 Bentall) and progressive NeoAR |
| Losay *et al.*^10^ | 2001 | 1,095 | 4.9±3.4 | NA | 13 (1.2) | NA | Progressive NeoAR in all patients |
| Losay *et al.*^11^ | 2006 | 1,156 | 6.25  (0-20) | 25 (2.1) | 16 (1.4) | Isolated AVR (9), Bentall procedure (4), AV-plasty (3) | NeoAR (15 >= Moderate, 1 Mild) and LV dilatation (16), Important NeoARD (4) |
| Ma *et al.*^12^ | 2016 | 583 | 3.83  (0.67-10) | 4 (0.7) | 3 (0.6) | AVR (3) | NeoAR and severe congestive heart failure or LVEF < 50%. |
| Martins *et al.*^14^ | 2018 | 127 | 7.4±4.7 | 24 (22.4) | 2 (1.6) | AVR (2) | Progressive NeoAR |
| Martins *et al.*^15^ | 2019 | 157 | 14.9±4.6 | 8 (5) | 1 (0.6) | Bentall procedure (1) | NeoAR and severe NeoARD |
| McMahon *et al.*^16^ | 2004 | 119 | 5.42  (1-15) | 3 (2.5) | 3 (2.5) | Aortic homograft (3) | Significant NeoAR with LV dilatation |
| Michalak *et al.*^18^ | 2013 | 172 | 13.5±2.4 | 28 (16) | 0 (0) | None | - |
| Nakayama *et al.*^21^*†* | 2019 | 469 | 19.0  (0.1-35.2) | 41 (8.6) | 17 (3.6) | AVR (11), AV plasty (3), Konno procedure (2), ARR (1) | Significant NeoAR |
| Oda *et al.*^22^ | 2012 | 387 | 10±7.4 | 29 (7.5) | 11 (2.8) | AVR (9), Bentall procedure (2) | Significant NeoAR (9), NeoAR and severe NeoARD (2) |
| Prifti *et al.*^24^ | 2002 | 134 | 3.4 (0.67-12) | 3 (3.2) | 2 (1.5) | AV plasty (1), Aortic homograft (1) | NA |
| Puras *et al.*^25^ | 2014 | 155 | 6  (0-25) | 15 (11) | 1 (0.6) | AVR (1) | Significant NeoAR |
| Schwartz *et al.*^26^ | 2004 | 335 | >5 (0-18) | 17 (5.1) | 8 (2.4) | NA | NA |
| van der Palen *et al.*^27^ | 2019 | 345 | 12.2 (1-39) | 33 (9.6) | 9 (2.6) | AVR (2), Switchback procedure (1), Bentall procedure (6) | NeoAR (5) and NeoARD (9) |
| W.K. Jhang *et al.*^28^ | 2012 | 240 | 6.6 (1-19.5) | 6 (2.4) | 2 (0.8) | AVR and -plasty (2) | Significant NeoAR |
| Walter *et al.*^29^ | 2010 | 324 | 14.4  (1-17.8) | 2 (0.6) | 2 (0.6) | AVR (2) | Significant NeoAR with LV dilatation and reduced LV function |
| Wang *et al.*^30^ | 2022 | 185 | 7.4 (0-15.6) | 19 (11.4) | 4 (2.2) | AV-plasty (2), AVR (1), ARR (1) | NeoAR or NeoARD |

*Significant NeoAR defined as >= Moderate NeoAR. †included in Figure 2B.

AV, aortic valve; AVR, aortic valve replacement; ARR, aortic root replacement; NeoAVS, Neoaortic valve surger

**Supplemental Table 4. Potential risk factors associated with occurrence of NeoAR at follow-up.**

| Variables |
| --- |
| Demographics |
| - Age at ASO^7,11,21,26,29^ |
| - Weight at ASO^30^ |
| - Sex^24^ |
| - Duration of follow-up^1,2,8^ |
| Associated lesions |
| - VSD^1,2,7,11,15,24,26,27,29^ |
| - TBA^7,11,16,31^ |
| - DORV^2,7,26^ |
| - AAA^8,11,15,24,29^ |
| - BPV^5,6,15^ |
| - LVOTO^2,7,22^ |
| - Non-facing commisures^17,18^ |
| Procedural characteristics |
| - Preoperative PR^21^ |
| - Ao/PA size discrepancy^1,8,11,12,14,17,30^ |
| - Trap-door coronary relocation^3,8^ |
| - Concomitant LVOTO relief^21,26^ |
| - Prior PAB^7,11,12,21,24,26,29,30^ |
| - Any NeoAR at discharge^7,9,11,21,29,30^ |
| Follow-up measurements |
| - Neoaortic root dilatation^1–3,5,8,16,27,28^ |
| - Aortic angle^15^ |

AAA, Aortic arch anomalies; Ao/PA, aorta / pulmonary artery; ASO, arterial switch operation; BPV, bicuspid pulmonary valve; DORV, double outlet right ventricle; LVOTO, left ventricular outflow tract obstruction; NeoAR, neoaortic regurgitation; PAB, pulmonary artery banding; PR, pulmonary regurgitation; TBA, Taussig-Bing anomaly; VSD, ventricular septal defect.

**Supplemental References**

1. Bové T, De Meulder F, Vandenplas G, et al. Midterm assessment of the reconstructed arteries after the arterial switch operation. *Ann Thorac Surg*. 2008;85(3):823-830. doi:10.1016/j.athoracsur.2007.10.043

2. Co-Vu JG, Ginde S, Bartz PJ, Frommelt PC, Tweddell JS, Earing MG. Long-term outcomes of the neoaorta after arterial switch operation for transposition of the great arteries. *Ann Thorac Surg*. 2013;95(5):1654-1659. doi:10.1016/j.athoracsur.2012.10.081

3. Formigari R, Toscano A, Giardini A, et al. Prevalence and predictors of neoaortic regurgitation after arterial switch operation for transposition of the great arteries. *J Thorac Cardiovasc Surg*. 2003;126(6):1753-1759. doi:10.1016/s0022-5223(03)01325-4

4. Hutter PA, Thomeer BJ, Jansen P, et al. Fate of the aortic root after arterial switch operation. *Eur J cardio-thoracic Surg Off J Eur Assoc Cardio-thoracic Surg*. 2001;20(1):82-88. doi:10.1016/s1010-7940(01)00752-7

5. Irwin M, Binney G, Gauvreau K, Emani S, Blume ED, Brown DW. Native Bicuspid Pulmonary Valve in D-Loop Transposition of the Great Arteries: Outcomes of the Neo-Aortic Valve Function and Root Dilation After Arterial Switch Operation. *J Am Heart Assoc*. 2021;10(18):e021599. doi:10.1161/JAHA.121.021599

6. Jeon B, Choi ES, Kwon BS, et al. The impact of a bicuspid pulmonary valve in the aortic position after arterial switch for transposition of the great arteries on neoaortic root dimension and function: a propensity score matched analysis. *Interact Cardiovasc Thorac Surg*. 2022;34(6):1106-1112. doi:10.1093/icvts/ivac073

7. Lange R, Cleuziou J, Hörer J, et al. Risk factors for aortic insufficiency and aortic valve replacement after the arterial switch operation. *Eur J cardio-thoracic Surg Off J Eur Assoc Cardio-thoracic Surg*. 2008;34(4):711-717. doi:10.1016/j.ejcts.2008.06.019

8. Lim HG, Kim WH, Lee JR, Kim YJ. Long-term results of the arterial switch operation for ventriculo-arterial discordance. *Eur J cardio-thoracic Surg Off J Eur Assoc Cardio-thoracic Surg*. 2013;43(2):325-334. doi:10.1093/ejcts/ezs264

9. Lo Rito M, Fittipaldi M, Haththotuwa R, et al. Long-term fate of the aortic valve after an arterial switch operation. *J Thorac Cardiovasc Surg*. 2015;149(4):1089-1094. doi:10.1016/j.jtcvs.2014.11.075

10. Losay J, Touchot A, Serraf A, et al. Late Outcome After Arterial Switch Operation for Transposition of the Great Arteries. *Circulation*. 2001;104(suppl_1):I-121-I-126. doi:10.1161/circ.104.suppl_1.I-121

11. Losay J, Touchot A, Capderou A, et al. Aortic valve regurgitation after arterial switch operation for transposition of the great arteries: incidence, risk factors, and outcome. *J Am Coll Cardiol*. 2006;47(10):2057-2062. doi:10.1016/j.jacc.2005.12.061

12. Ma K, Li S, Hu S, et al. Neoaortic Valve Regurgitation After Arterial Switch: Ten Years Outcomes From A Single Center. *Ann Thorac Surg*. 2016;102(2):636-642. doi:10.1016/j.athoracsur.2016.02.037

13. Marino BS, Wernovsky G, McElhinney DB, et al. Neo-aortic valvar function after the arterial switch. *Cardiol Young*. 2006;16(5):481-489. doi:10.1017/S1047951106000953

14. Martins CN, Gontijo Filho B, Lopes RM, Silva F das CLE. Mid- and Longterm Neo-Aortic Valve Regurgitation after Jatene Surgery: Prevalence and Risk Factors. *Arq Bras Cardiol*. 2018;111(1):21-28. doi:10.5935/abc.20180111

15. Martins D, Khraiche D, Legendre A, et al. Aortic angle is associated with neo-aortic root dilatation and regurgitation following arterial switch operation. *Int J Cardiol*. 2019;280:53-56. doi:10.1016/j.ijcard.2019.01.042

16. McMahon CJ, Ravekes WJ, Smith EO, et al. Risk factors for neo-aortic root enlargement and aortic regurgitation following arterial switch operation. *Pediatr Cardiol*. 2004;25(4):329-335. doi:10.1007/s00246-003-0483-6

17. Michalak KW, Moll JA, Moll M, Mludzik K, Moll JJ. Neoaortic valve function 10 to 18 years after arterial switch operation. *World J Pediatr Congenit Heart Surg*. 2010;1(1):51-58. doi:10.1177/2150135110361361

18. Michalak KW, Moll JA, Moll M, et al. The neoaortic root in children with transposition of the great arteries after an arterial switch operation. *Eur J cardio-thoracic Surg Off J Eur Assoc Cardio-thoracic Surg*. 2013;43(6):1101-1108. doi:10.1093/ejcts/ezs709

19. Michalak KW, Sobczak-Budlewska K, Moll JJ, et al. Neoaortic Regurgitation in Patients with Transposition Long Term After an Arterial Switch Operation and Its Relation to the Root Diameters and Surgical Technique Used. *Pediatr Cardiol*. 2020;41(1):31-37. doi:10.1007/s00246-019-02217-w

20. Muneuchi J, Watanabe M, Sugitani Y, et al. Being Overweight Is Related to Neoaortic Sinus Dilatation After Arterial Switch Operation. *Texas Hear Inst J*. 2022;49(5):e207508. doi:10.14503/THIJ-20-7508

21. Nakayama Y, Shinkawa T, Matsumura G, Hoki R, Kobayashi K, Niinami H. Late Neo-Aortic Valve Regurgitation Long After Arterial Switch Operation. *Ann Thorac Surg*. 2019;108(4):1210-1216. doi:10.1016/j.athoracsur.2019.04.009

22. Oda S, Nakano T, Sugiura J, Fusazaki N, Ishikawa S, Kado H. Twenty-eight years’ experience of arterial switch operation for transposition of the great arteries in a single institution. *Eur J cardio-thoracic Surg Off J Eur Assoc Cardio-thoracic Surg*. 2012;42(4):674-679. doi:10.1093/ejcts/ezs033

23. Oda S, Nakano T, Fujita S, Sakaguchi S, Kado H. Long-Term Growth of the Neoaortic Root After Arterial Switch Operation. *Ann Thorac Surg*. 2019;107(4):1203-1211. doi:10.1016/j.athoracsur.2018.09.025

24. Prifti E, Crucean A, Bonacchi M, et al. Early and long term outcome of the arterial switch operation for transposition of the great arteries: predictors and functional evaluation. *Eur J cardio-thoracic Surg Off J Eur Assoc Cardio-thoracic Surg*. 2002;22(6):864-873. doi:10.1016/s1010-7940(02)00613-9

25. Rodríguez Puras MJ, Cabeza-Letrán L, Romero-Vazquianez M, et al. Mid-term morbidity and mortality of patients after arterial switch operation in infancy for transposition of the great arteries. *Rev Esp Cardiol (Engl Ed)*. 2014;67(3):181-188. doi:10.1016/j.rec.2013.06.021

26. Schwartz ML, Gauvreau K, del Nido P, Mayer JE, Colan SD. Long-term predictors of aortic root dilation and aortic regurgitation after arterial switch operation. *Circulation*. 2004;110(11 Suppl 1):II128-32. doi:10.1161/01.CIR.0000138392.68841.d3

27. van der Palen RLF, van der Bom T, Dekker A, et al. Progression of aortic root dilatation and aortic valve regurgitation after the arterial switch operation. *Heart*. 2019;105(22):1732-1740. doi:10.1136/heartjnl-2019-315157

28. Jhang WK, Shin HJ, Park JJ, et al. The importance of neo-aortic root geometry in the arterial switch operation with the trap-door technique in the subsequent development of aortic valve regurgitation. *Eur J cardio-thoracic Surg Off J Eur Assoc Cardio-thoracic Surg*. 2012;42(5):794-799; discussion 799. doi:10.1093/ejcts/ezs169

29. Delmo Walter EM, Huebler M, Alexi-Meshkishvili V, Sill B, Berger F, Hetzer R. Fate of the aortic valve following the arterial switch operation. *J Card Surg*. 2010;25(6):730-736. doi:10.1111/j.1540-8191.2010.01144.x

30. Wang Z, Li Z, Ding N, et al. Incidence and risk factors for late complications after the arterial switch operation. *J Card Surg*. 2022;37(10):3117-3132. doi:10.1111/jocs.16745

31. van der Palen RLF, van der Zee C, Vink AS, et al. Transposition of the great arteries: Fetal pulmonary valve growth and postoperative neo-aortic root dilatation. *Prenat Diagn*. 2019;39(12):1054-1063. doi:https://doi.org/10.1002/pd.5539
